# Developing a Mediterranean Healthy Food Basket and an Updated Australian Healthy Food Basket Modelled on the Australian Guide to Healthy Eating

**DOI:** 10.3390/nu15071692

**Published:** 2023-03-30

**Authors:** Ella L. Bracci, Courtney R. Davis, Karen J. Murphy

**Affiliations:** Clinical and Health Sciences, Alliance for Research in Exercise, Nutrition and Activity, University of South Australia, GPO Box 2471, Adelaide, SA 5001, Australia

**Keywords:** healthy food basket, Australian dietary guidelines, Mediterranean diet, western diet, nutritional adequacy, food security

## Abstract

Introduction: Australian healthy food baskets are typically modelled off the Government Guidelines for healthy eating. However, these baskets have not been updated recently, nor has there been a Mediterranean Diet basket developed for an Australian population despite research suggesting high adherence is possible and subsequent health benefits observed. Food baskets typically only present the nutrition profile or the cost of a basket, seldom both. Methods: Baskets were developed based on the Australian Guide to Healthy Eating, The Mediterranean Diet and typical Australian dietary intake (Western Diet). Four reference families were created based on data from Australian censuses and population statistics. Seven-day meal plans for reference families were entered into Foodworks software and aimed to meet 100% of nutrition and energy requirements. Basket costs were calculated from Coles Australia online. Results: The AGHE basket met all NRVs except for VLCN3 for the 7-year-old male (73% adequate intake). The Mediterranean Diet met all NRVs except zinc (44-year-old male) ranging from 98 to 257% of the RDI. The Western Diet failed to meet NRVs for numerous nutrients. The MedDiet baskets were generally cheaper ($78 for a one-person household to $285 for a four-person household) than AGHE and Western Diet. Discussion: Meeting nutrition requirements over seven days for zinc can be challenging for males. Fortified products provide an opportunity to improve nutrient profile; however, nutrient intake should equilibrate over time. Further, cost saving strategies can increase affordability. This research suggests a MedDiet is not more costly than a typical Western Diet or healthy AGHE diet.

## 1. Introduction

Diet is one of the leading modifiable risk factors for chronic disease. However, data indicates that a significant proportion of Australians are not consuming a balanced healthy diet consisting of the five core food groups: fruit (1), vegetables and legumes (2), breads and cereals (3), dairy foods (4) and meat, and alternatives (5) recommended by the Australian Dietary Guidelines (ADG) and Australian Guide to Healthy Eating (AGHE) [1,2]. Consuming foods from these food groups can reduce the risk of several chronic diseases and health complications including type two diabetes, heart disease, obesity, and osteoporosis.

According to the 2017–2018 National Health Survey, only 8% of Australians consume the recommended 375 g of vegetables per day [2]. However, the average Australian consumes up to 35% of energy from discretionary foods high in salt, added sugars, and unhealthy fat, reflective of a Western Diet [2].

Reflective of these dietary trends, obesity rates in Australia have risen, with almost two thirds of the population experiencing obesity, costing the economy $11.8 billion in 2018 [3]. The obesity-related trends are considered reflective of the Western Diet with high amounts of discretionary foods that are energy dense but low in nutritive value, in combination with high amounts of unhealthy saturated fats and added sugars from processed foods. To tackle the issues with population intake, globally evidence-based recommendations for healthy eating have been steered towards plant-based diets lower in saturated fats, animal products, and added sugars. The Mediterranean Diet has increased in popularity due to its known health benefits, such that it was endorsed by the American Dietary Guidelines (2015–2020) [4]. Research suggests that Australian populations can adhere long-term to a Mediterranean style eating pattern and improve their health [5,6,7,8]; however, individuals may need guidance from nutrition professionals to modify the Mediterranean Diet to meet their needs and preferences in an Australian context and adhere long-term (>1 year) [7].

Though a range of factors impact dietary choice, one explanation for the low intake of ‘healthy’ foods may relate to the misconception that purchasing such foods and beverages is too expensive and unaffordable [9,10,11,12]. However, previous research implies this is not necessarily the case within Australia [13]. Though some price discrepancies may exist between differing socioeconomic and regional areas, generally ‘healthy’ nutrient-dense foods such as fruit and vegetables are priced affordably and are non-taxable (Government Services Tax-Free) [11,12,13,14]. However, recently, the impact of the global COVID-19 pandemic, inflation, the cost of living, and natural disasters has impacted cost and access to healthy foods. Data alarmingly suggests that up to 21% of Australians may be experiencing food insecurity and the demographic of people utilizing food relief has extended to include international students and casual workers [15,16].

Australian food baskets have been developed as early as 2001 [17] in hopes to monitor food affordability and food pricing. These food baskets are typically developed by nutrition professionals and researchers in various Australian states and based on the Australian Dietary Guidelines and Australian Guide to Healthy Eating [18]. However, some food baskets previously developed no longer align with the current housing and family composition trends, namely multi-generational households, and may need to be updated to reflect this change. Additionally, baskets typically aim to meet between 70 and 95% of either individual nutrition requirements, or household requirements overall for a 7- or 14-day period rather than 100% of nutrition requirements. That being said, some have previously sought to meet 100% of nutrition requirements. To our knowledge, a Mediterranean-styled food basket has never been developed for an Australian population, and could be beneficial considering many global dietary guidelines are moving towards the adoption of predominantly plant-based dietary patterns such as the Mediterranean Diet. One explanation for the lack of development of MedDiet baskets could be due to the perceived cost of following a MedDiet considering it contains expensive components such as extra virgin olive oil, oily fish, and nuts. International researchers have used Mediterranean Diet food modelling, but typically in the context of reducing greenhouse gas emissions (CO_2_, CH_4_, NH_3,_ and N_2_O) and improving the environmental impact through dietary choices aligning with a Mediterranean Diet [19,20], and not in the context of food baskets. Therefore, we aim to address this gap and provide greater feasibility of following a MedDiet in Australia in terms of cost and nutritional value.

Differing methodologies also exist with calculating the costs of these food baskets which can cause difficulty with comparability across baskets. As identified by Lewis and Lee [21], such differences include the use of 12 different pricing tools and different geographic levels including local government and regional areas [21]. Basket costs may be calculated based on different locations such as major cities, inner and outer regional, remote, and very remote [22,23]; low, medium, or high socioeconomic areas [24,25]; or metropolitan areas [26], and include large supermarket chains, greengrocers, butchers, and/or small independently owned businesses [22,26].

Further, the food baskets may focus specifically on the cheapest possible basket including only home-brand items, which may not reflect true consumer purchasing trends [21], while others collect the price of an item from up to three different brands to calculate the average, or record the cheapest brand item, excluding the home-brand or generic item [22,25,26]. Moreover, previous food baskets may be somewhat lost in translation considering they typically tend to report grocery style lists but fail to provide a meal plan or meal guide as to how the foods and beverages within the list can be consumed throughout the 7- or 14-day period [24,27].

### Aims

With the array of differing food basket methodologies, we aim to simplify such methodologies and present two nutritionally adequate and affordable food baskets with a comparator basket reflective of Australian’s current dietary trends.

Firstly, reference families were determined based on current housing and population data to reflect four ‘average’ Australian households and account for differing nutritional requirements across age and gender.

Secondly, two food baskets were created to meet 100% of nutrition and energy requirements for the reference families and individuals over 7 days to demonstrate the ability to meet such requirements with a shorter length of dietary modelling, expecting that nutrient levels would certainly level out over 14 days. One basket was modelled on the Australian Guide to Healthy Eating, while the other was developed based on the Mediterranean Dietary pattern. Meal plan guides are presented to conceptualize how the basket could be utilized in conjunction with the standard grocery style list presented in other food baskets to improve translation.

Thirdly, the costs of the baskets were determined using a simple previously published methodology, namely utilizing Coles Australia online grocery shopping using a mix of home-brand and branded items to reflect true consumer trends. The food baskets were assessed in relation to food security data to determine affordability.

Lastly, a Western Diet meal plan was created based on Australian’s reported dietary intake to act as a comparator in terms of nutritional quality and cost.

## 2. Methodology

### 2.1. Developing Reference Families to Represent Australian Households

Four reference households were developed to reflect a range of nutritional needs of Australians (Table 1). The composition of these households was informed using a combination of 2012–2013 and 2015–2016 data from the Australian Bureau of Statistics Family Characteristics Survey [28] and the Census of Population and Housing data [29] to identify the most common age, gender, and household type in Australia.

#### 2.1.1. Households

During 2016, almost three quarters of households were ‘family’ households (71%), and one quarter (24%) were single- or lone-person households [29]. During 2012–2013, 74% of the 8.9 million households were family households, 23% were lone- or single-person households, and the remaining 3% were group households. These data indicate that the households do not tend to differ by large percentages between censuses. Additionally, most lone dwellers (52%) were females during 2012–2013.

#### 2.1.2. Families and Family Characteristics

According to 2016 census data, 45% of families in Australia were couple families with children, while 38% were couples without children [29]. Single parent families accounted for 16% of families, 82% of which were female single parents, and the remaining 2% of families were “other families” [29]. However, during 2012–2013, families with children made up 58% of all families, 78% of which had dependent children (0–17 years of age), and 22% had non-dependent children (≥18 years old). In couple families, 23% of children were aged 0–4 years old, 21% were aged 5–9 years old and 20% were aged 10–14 years old. The remaining percentages broadly described dependent students aged 15–24, non-dependent students aged 15–24 and, 25 years and over (17%, 11%, and 7%, respectively) [28]. In one-parent families, children aged 0–4 accounted for 12% of total children, 5–9-year-olds accounted for 17%, 10–14-year-olds accounted for 19%, and the remaining 53% of total children were dependent students and non-dependent students aged 15–24 as well as those aged 25 years and over [28].

#### 2.1.3. Reference Families for the Healthy Food Baskets

Four reference families were devised based on census data and family characteristic data to allow for a variety of nutritional needs and income (Table 1). Household 1 comprises a couple family with both adults aged 44 and two children, a 7-year-old male and 15-year-old female. According to the 2016 Census of Population and Housing, 3.36 million adults in households were aged between 25 and 34 years old, and 3.14 million persons were aged between 35 and 44 years-old [28]. As the nutritional requirements for an adult typically do not change substantially between the ages of 19 and 50 years old, the upper age of 44 was selected despite more persons aged between 25 and 34 years old. The dependent children within the couple family were selected in a similar pragmatic manner. The Family Characteristics survey indicated that, combined, most children were aged 0–14 years old (64%). The midpoint, a 7-year-old child was selected. The second child in the couple family was determined to be a 15-year-old female due to the increased requirements for multiple nutrients including iron, magnesium, folate, and riboflavin within the 14–18-year-old age group.

Household two is a single mother with the same characteristics and child characteristics as household one (Table 1).

Household three comprises two elderly pensioners, both aged 70 years. Almost two thirds (64%) of all Australian adults living in private dwellings were married or in a registered marriage during 2012–2013 [28]. Additionally, a high proportion of those aged 75 and over (51%) were married at the time of census [28]. Therefore, an elderly couple was selected as opposed to a lone pensioner. Though a higher number of older adults were aged 55–64-years-old (2.75 million) compared to the 65–74-year-old age group (2.08 million), the nutrition requirements change at ≥70-years-old. Additionally, those aged 55–64 do not receive the pension, nor are they necessarily retired, which is a focal point for this household due to the impact of food security and lower income in vulnerable groups.

Household four consists of a single adult, namely a 30-year-old female. A higher proportion of females were lone dwellers as indicated by both the census and family characteristics survey. As nutrition requirements do not differ for females aged 19–50, except for magnesium (10 mg difference), the midpoint between 25 and 34 was selected.

### 2.2. Calculating Income for Reference Families

The average weekly income for Australians was used as a reference point. The average weekly income for the four households was calculated under the assumption that all adults, apart from the elderly pensioner household, were working full time (38 h per week). In 2021, 7 million people were working full time and half (4 million) were working part time [30].

The average weekly earnings (gross) for the reference period of May 2022 for males and females working full time was AUD$1835 (seasonally adjusted) (Table 2) [31]. Despite some disturbances to businesses due to the ongoing COVID-19 pandemic, this reference period is considered reflective of a stable workforce similar to that prior to COVID-19 [31].

This average was used for HH1 and HH3 and multiplied for HH1 (Table 2). An assumption was made that the 15-year-old was not working nor contributing to household income.

Average weekly income for HH3 was calculated using age pension rates under the assumption that the elderly couple meets the requirements for the pension including age (66 years and 6 months), residence rules, income test, and assets test. Further, presumably, this household would only be receiving the ‘standard’ pension, as opposed to a veteran or disability pension where the payments differ. Additionally, the weekly income is based on pension income only (base, supplement, and energy supplement) and excludes any potential superannuation income or investments that may be accruing interest.

### 2.3. Creating and Analysing Meal Plans

A 7-day meal plan was created in Foodworks 10 dietary software Version 10 (Xyris, Brisbane, Australia) for the three food baskets (AGHE, MedDiet and Western Diet). Each household had a separate Foodworks file. All meal plans were reviewed by two dietitians to ensure the meal plans fit the relevant diet compositions.

#### Meal Plan Requirements and Parameters

The AGHE and MedDiet meal plans had specific parameters that needed to be met as follows:

- Meets 100% of nutrition requirements and energy requirements (RDI, AMDR);
- Meets food group recommendations;
- Provides < 10% of total energy from saturated fat;
- Favourable fat ratio Polyunsaturated:Monounsaturated:Saturated (P:M:S);
- Adequate intake of omega-3-fatty acids (Eicosapentaenoic acid, Docosapentaenoic acid and Docosahexaenoic acid).

Recommended Daily Intake (RDI) refers to the level of intake essential to meet the nutritional needs of most healthy people (96%), and was the preferred value when assessing nutrient intake. Where an RDI was not available, Adequate Intake (AI), Upper Level of Intake (UL), and Suggested Dietary Targets (SDTs) were used.

Though the Western Diet food basket had no specific nutrition targets or parameters, the meal plan aimed to align with Australian’s reported intake of food group, portion sizes, and daily energy from the 2011–2012 Australian Health Survey.

### 2.4. Energy Requirements for Households

Self-reported height and weight data from National Health Surveys were not used to calculate energy requirements due to the prevalence of under and over reporting; however, they were used to guide the Western Diet basket. Estimates from the National Health Survey (2004–2005) reportedly have a relative standard error of 25–50% or ≥50% (Table 3) [34]. Energy requirements were derived from references values, the Eat for Health energy calculator, and from the summation of the recommended servings of food groups per day based on the upper value of their energy contribution [18].

#### 2.4.1. Older Adults and Adults

The reference energy level of 8700 kJ/day (2071 kcal/day) was used for adults aged 19–50 [35,36]. This reference value is an approximate energy guide for Australians to maintain weight and is often displayed on food and beverage product labels to provide nutrition information to consumers. The energy intake (8700 kJ/day) is based on the average intake required for a sedentary (PAL 1.4) male (9900 kJ/day) and female (7600 kJ/day) [18]. Additionally, the NHMRC Nutrient Reference values for dietary energy (31–50-year-olds) and the Eat for Health daily energy requirements calculator were consulted to check energy values for the 44-year-old male and female for HH1 and HH2 and similarly fell between 8700 kJ/day and 8900 kJ/day [18].

The NHMRC Nutrient Reference values for dietary energy (51–70-year-olds) was utilized to determine an approximate daily energy intake for the 70-year-old female and male in HH3. An average daily energy intake was calculated for males and females with a physical activity level (PAL) of 1.4 across the different heights and weights available (BMI 22 kg/m^2^). An average energy intake of 8400 kJ/day (2000 kcal) was calculated. When consulting the daily energy requirements calculator, the approximate energy intake for a male and female aged 70 with a PAL of 1.4 was 8900 kJ/day (2120 kcal/day). As energy ranged from 8400 kJ to 8900 kJ, the average of 8700 kJ was used for the 70-year-old male and female in addition to the 40-year-old male and female.

#### 2.4.2. Children and Adolescents

The NHMRC Nutrient Reference values for dietary energy was referred to for the 15-year-old female and 7-year-old male in HH1 and HH2. The estimated energy requirements (EER) are calculated using basal metabolic rate predicted from a reference height and weight. As the EER only provides one reference weight and height per age (3–18-years old), the Eat For Health daily energy requirements calculated was also utilized. The 15-year-old female required approximately 8200 kJ/day (1950 kcal/day) based on a weight and height of 52 kg and 1.62 m, respectively. The energy calculator yielded a similar result of 8130 kJ/day (1936 kcal/day). The 7-year-old male required approximately 6100 kJ/day (1450 kcal) based on a weight and height of 23 kg and 1.22 m, respectively. Similarly, the energy calculator predicted energy requirements to be approximately 6010 kJ/day (1430 kcal/day).

However, we opted to calculate the energy requirement based on the recommended number of servings per day of the five food groups for a 15- (Table S1) and 7-year-old (Table S2) and their energy contribution, as this is a period of growth and development for children and adolescents where energy should not be restricted or underestimated. The upper value was used, i.e., a serving of vegetables ranges from 100 to 350 kJ. The calculated energy for all household members was compared against Australian’s reported daily energy intake (Table 3). Data from the 2011–2012 Australian Health Survey indicated the approximate energy intake of Australians, which was taken into consideration when developing the Western Diet basket. However, it is acknowledged that under-reporting energy intake is an issue.

##### Assumptions

Across the three food baskets, assumptions were made, including that the male and female in couple households would be eating similar meals. Further, the children in in HH1 and HH2 will eat most of the same main meals but with different portions and some modifications, i.e., dinner meals. Additionally, there is the assumption that household members may eat slightly differently on weekend days, leftovers from main meals will be utilized as subsequent meals, i.e., dinner leftovers for lunch, and some meals will be pre-packaged (pre-prepared) or not prepared in the home. Moreover, in all food baskets, there was the assumption that no household members had any food allergies, intolerances, or aversions. A combination of hot and cold dinners and lunches, i.e., soups and salads, were used to account for seasonality and variety rather than developing meal plans based on a single season of the year.

### 2.5. Australian Guide to Healthy Eating (AGHE) Meal Plan

The Australian Guide to Healthy Eating (AGHE) meal plan was based on the Australian Dietary Guidelines and the AGHE including the recommended daily number of servings per food group based on age, gender, and life stage [18]. Additional considerations included at least two thirds of grains were wholegrains, meats and processed meats had <10% fat, dairy products were mostly reduced fat, and the meal plan adhered to the acceptable macronutrient distribution of 15–25% protein, 45–65% carbohydrate, and ≤35% fat.

Example meals from the Eat for Health website [18] were utilised to assist in creating the meal plan, and modifications were made to meet the required levels of vitamins, minerals, trace elements, and energy requirements (Supplementary Tables S3 and S6).

### 2.6. Mediterranean Diet Meal Plan

The Mediterranean Diet meal plan was developed to include the foundational principles of a MedDiet including large amounts of extra virgin olive oil, fruit, vegetables, legumes, nuts, seeds, moderate amounts of fish, poultry, dairy foods, and low amounts of red meat and processed foods [37,38].

The literature review by Davis, Bryan [38] provided a reference point for the quantity (grams and servings per day) of Mediterranean diet food groups and frequency of which they are recommended to be consumed across a typical week. For example, in addition to recommended daily servings of fruit, vegetables, grains, dairy foods, and extra virgin olive oil, there are weekly targets for red meat (≤1 serve per week), seafood (3+ servings per week), legumes (3+ servings per week), and nuts (5+ servings per week) in which the meal plan took into consideration. Further, the MedDiet meal plan was designed to meet Australian NRV’s, namely calcium, iron, and zinc, and developed to meet an approximate 40:40:20 macronutrient distribution of carbohydrate, fat, and protein. Additionally, a guideline for the intake of fatty acids was derived from the Davis et al. literature review [38]. Example meals were gathered from both recipe books and meal plan templates from previous dietitian led Mediterranean Diet randomized controlled trials and tailored to meet 100% of nutrition and energy requirements [39] (Supplementary Tables S4 and S7). Where recipes were used, they were modified to be a single serve to ensure accuracy in calculating the quantity of ingredients required.

Due to the nature of the Mediterranean Diet having large amounts of nutrient and energy-dense nuts, seeds, and EVOO, an ‘allowance’ of a supplementary 1000–1500 kJ in addition to daily energy requirements were anticipated, i.e., for adults aged 19–50 requiring approximately 8700 kJ, energy could range from 8700 to 10,200 kJ on a MedDiet. Further, though a traditional Mediterranean Diet includes a weekly intake of red wine, this was excluded in the current research to align with previously published healthy food baskets which historically do not include alcoholic beverages.

### 2.7. Western Diet Meal Plan

The Western Diet comparator meal plan was based on Australian dietary intake data from the most contemporary Australian Health Survey (AHS) in 2011–2012 [1]. According to the AHS, less than 4% of Australians (aged 2+ years-old) met the recommendations for the number of servings of vegetables per day, and only one third (31%) met the fruit recommendations [1]. First results and key statistics from the 2017–2018 National Health Survey (NHS) indicated a substantial increase in those meeting fruit recommendations (51% vs. 31%) compared to 2011–2012, but a negligible increase in those meeting vegetable recommendations (8% vs. 4%) was observed [2]. Similarly, Australians were unlikely to meet the recommendations for the remaining food groups [2], which was reflected in the meal plan.

Though Australians are not meeting their recommended servings for the core five food groups, discretionary servings are overconsumed, with approximately 30–35% of energy coming from discretionary foods (equivalent of six serveings per day). Due to the high number of discretionary foods within a Western Diet, it was assumed that the most pragmatic approach would be to include regular fat, sugar, and salt products, as well as a majority of white processed breads and cereals. Average servings of the five food groups per day, discretionary foods, and intake of spreads and oils was extrapolated from the Australian Health Survey (2011–2012) to guide the meal plan in addition to reported daily energy intake [1]. Additionally, the types of common foods from each food group reported in the AHS were used to form the meal plan based on Australian’s dietary choices and intake (Supplementary Tables S5 and S8).

### 2.8. Calculating the Cost of Meal Plans and Food Basket

Foodworks databases for each household were exported into Microsoft Access separately. Foods from the ‘DocFoods’ MS Access table were sorted alphabetically to display where the same food or beverage was consumed over the week. Once alphabetized, each food or beverage and the weekly quantity was recorded into a Microsoft Excel spreadsheet. A separate Excel spreadsheet was created for the AGHE, the MedDiet, and the Western Diet meal plans with different excel sheets for HH1, HH2, HH3, and HH4. Foods and beverages were assigned a code depending on their food group and classification, i.e., 1 = fruit, 2 = vegetables, 3 = breads, grains cereals, 4 = lean meat, poultry, alternatives, 5 = dairy and alternatives, 6 = other, and 7 = discretionary. Foods and beverages were subsequently sorted by code to keep similar products together and calculate the cost per food group. When calculating the cost contribution for each of the food groups including fruit, vegetables, grain foods, meat and alternatives, dairy foods and discretionary, certain specifications were made. Namely, tomatoes and avocadoes were allocated to the vegetable group despite their botanical classification (fruit) to align with previous research [22,40,41]. Additionally, legumes and beans contribute to both servings of vegetables and meat alternatives; however, they were allocated to vegetables in the current research [22,40].

Prices for items from meal plans were sourced from Coles Australia Online as per previously methodology by the authors [40] during October–November 2022 using post code 5000 (CBD, Adelaide, South Australia). Recorded prices were standard, not discount or ‘price-locked’ prices, for consistency and to accommodate for fluctuating seasonal prices. A combination of generic or home-brand items were included in combination to popular brands, i.e., Vegemite and Weet-Bix, in line with previous healthy food baskets [25,26]. Brand names including home-brand, unit size (g, kg, mL, L), and unit weight were recorded into excel spreadsheets. Spices and herbs were not included as per previous food baskets, but honey, oil, and additional ‘non-core’ foods were included.

To assess affordability of the food baskets, the average weekly income calculated previously (Section 2.2) was used to determine the percentage of income required to purchase the basket for each household. Higher proportions of income required are considered less favourable, particularly for lower income individuals and families, whereby other categories of spending (housing and repayments, fuel, power, debts) take precedent over money dedicated to groceries. Research suggests up to 30% of disposable income can be required for a ‘healthy diet’ which indicates a balance of the five food groups with occasional discretionary foods [13]. Spending greater than 30% of disposable income could potentially put Australians at risk of food insecurity [9,13,42,43].

## 3. Results

### 3.1. Nutritional Adequacy of Baskets

#### 3.1.1. Food Groups, Energy, and Macronutrients

##### AGHE

Energy and macronutrients

Overall energy requirements were met or exceeded by all household members (Table 4). However, the 70-year-old female and 15-year-old female were between 400 and 800 kJ under the energy requirement. The percentage of daily energy attributed to carbohydrate ranged from 42 to 47% energy, with the 70-year-old male having the lowest contribution of daily energy from carbohydrate and the 7-year-old male having the highest. All individuals had <30%en from fat with <10%en from saturated fat. Further, %en from protein from all individuals met guidelines of 15–25% and ranged from 22 to 24% (Table 4).

**Food Groups**

All household members met and exceeded fruit and vegetable recommendations ranging from 2.1 to 2.7 servings and 5.2 to 6.5 servings, respectively (Table 4). Additionally, grain servings were met and exceeded by all household members and provided up to four additional servings (70-year-old female). The recommended servings for dairy and meat and alternatives were met, with most individuals having an extra 0.5 to 1.5 servings, and discretionary servings were within the recommended range for all individuals (Table 4).

##### MedDiet

**Energy and macronutrients**

Average daily energy intake ranged from 7634 kJ to 9859 kJ for household members, meeting and exceeding calculated requirements (Table 5). Percentage of daily energy intake from carbohydrate and fat ranged from 37 to 39%, while protein accounted for 18–20% of daily energy. All household members had <10% daily energy from saturated fat.

**Food Groups**

All household members were within the recommended range for daily intake of fruit and vegetables, ranging from 2.0 to 2.9 and 5.1 to 8.3 servings per day, respectively (Table 4). Additionally, daily servings of grain were within the range (4 to 6 servings per day) for the 7M and 70F, while the remaining household members exceeded the recommendation range of 6.6 to 8.5 servings per day. Dairy servings were met and exceeded by all household members ranging from 2.1 (44M) to 3.3 (15F), and daily virgin olive oil allowance was met except for the 7M who had half a tablespoon less than recommended (Table 4).

Weekly red meat intake ranged from 0.7 to 1.1 servings, with certain household members slightly exceeding the recommendation (44M and 15F). On the other hand, weekly servings of nuts were only met by 2 household members, with servings ranging from 2.7 to 6.0. Weekly legumes and seafood servings were met by all household members except the 7M, ranging from 3.0 to 5.5 servings and 1.4 to 7.4 servings, respectively (Table 5). The 7M met less than half (47%) of the seafood recommendation (three servings per day). Discretionary foods were within range (0–2 servings) for all household members.

##### Western Diet

Energy and macronutrients

Daily energy intake ranged from 7800 kJ to 9958 kJ for household members, with the 7M having the lowest intake and the 44M having the highest (Table 6). Carbohydrates contributed between 43 and 52% of daily energy, while protein and fat ranged from 12 to 15% and 35 to 40%, respectively, and were either below or above recommendations. Saturated fat ranged from 14 to 20% of daily energy, exceeding the ≤10% recommendation (Table 6).

**Food Groups**

No household member met the daily recommended servings for fruit, vegetables, grain, dairy foods or meat, or alternatives except the 7M, who met fruit intake (1.6 servings per day), and the 70F and 70M, who were within the range for grain foods (4.3 to 4.5 servings per day). Household members’ fruit and vegetable intake ranged from 1.1 to 1.6 servings per day and 1.4 to 3.4 servings per day, respectively. Household members were consuming less than 4.8 servings of grain and 1.9 servings of dairy per day. Servings of meat and alternatives were also low, ranging from 0.8 to 2.2 servings per day, while discretionary foods exceeded recommendations by up to 4 times, ranging from 5.6 to 6.0 daily serves (Table 6).

#### 3.1.2. Nutrient Reference Value AGHE

All NRVs for vitamins and minerals were met (100%) by all household individuals (Table 7). Sodium intake ranged from 96 to 99% for the two adults (44-year-old) and 98 to 100% for the children (15-year-old female and 7-year-old male). Iron was just met (101%) for the 44-year-old female, while conversely, Zinc was just met for the 44-year-old male (101%). Similarly, linoleic acid just met recommendations (100–102%) for the two 70-year-old household members and the 44-year-old male and 7-year-old male (Table 7). Long-chain omega-3 fatty acids were exceeded by over seven times in the 7-year-old male (731% of the adequate intake), but only met 13% of the upper limit.

##### Mediterranean Diet

All NRVs for vitamins and minerals were met (100%) by all household individuals apart from the 44-year-old male who did not meet Zinc requirements (98%) (Table 8). Sodium levels ranged from 86 to 100% across individuals and households, with the 7-year-old male and 77-year-old male having the lowest average amount across the week. Iron was just met by the 44-year-old female (101%), while the 44-year-old male only just met linoleic and alpha-linolenic acid requirements (102% and 104%, respectively). The modified MedDiet was able to meet calcium requirements for all household individuals ranging from 118 to 180% of the RDI. VLCN3 was exceeded by almost six times in the 7-year-old male (596% of the adequate intake), but only met 11% of the upper limit (Table 8).

##### Western Diet

All NRVs for riboflavin, niacin, vitamin C, vitamin B12, folate, and alpha-linolenic acid were met by all household members (>100%) (Table 9). However, all household members were low in at least three important micronutrients. Fibre intake ranged from 52 to 74%, with the 44M having the lowest and 77F having the highest daily intake (Table 9). All household members were low in potassium and calcium, meeting 62–91% and 48–95%, respectively, and all household members exceeded sodium intake (121–205%). Thiamin intake for the 44M was less than recommended (86%), while vitamin E was low for the 15F (82%). Magnesium, selenium, vitamin B6, and VLCN3 intake were low in all household members except for the 7M ranging from 51 to 73%, 86 to 97%, 54 to 78% and 19 to 26%, respectively. Vitamin A recommendations were not met for the 77M, 44F, 44M, or 15F ranging from 67 to 78%, nor was iron for 44F, 44M, 15F, and 7M (38–96%). Both the 77M and 44M did not meet zinc requirements (72–75%) or linoleic acid requirements (77–80%) (Table 9).

### 3.2. Cost of Food Baskets

#### 3.2.1. Total Cost of Basket

The cost for the AGHE basket ranged from $75 to $315 across households compared to the cost of the MedDiet basket, which ranged from $78 to $285 and was generally cheaper across households (Table 10). Similarly, overall, the initial outlay for the MedDiet was cheaper ($220–$467) compared to the AGHE ($204–$481). However, the MedDiet basket was slightly more expensive for a single person (HH4) by $16. The Western Diet comparator cost between $80 to $313 across households, and required an initial outlay ranging between $217 and $504 per week (Table 10).

Percentage income required for the AGHE basket ranged from 4 to 24% across households when calculated on the weekly meal plan and 11 to 42% when calculated based on the initial outlay required to purchase basket items that would last beyond the week (Table 10). The households with the greatest percentage of income required were HH3 (24–42% weekly income) and HH2 (13–21% average income). The MedDiet basket similarly required a higher percentage of income for HH3 (17–34% weekly income) and HH2 (11–20% weekly income). HH1 and HH4 ranged from 8 to 13% and 4 to 12%. Likewise, HH3 and HH2 required higher proportions of income, 20–40% average income and 12–21%, respectively. Though the initial outlay for HH1 was an additional $23–$37, the percentage of income required was similar to that of the AGHE and MedDiet baskets (Table 10).

#### 3.2.2. Cost Per Food Group

##### AGHE

Meat and alternatives and vegetables generally contributed the most to the cost of the AGHE basket across households ranging from $21 to $74 and $19 to $70, respectively (Table 11). Dairy foods and grains contributed similar amounts, ranging between $11 and $45 and $6 and $44. Fruit generally contributed half that of vegetables and meat and alternatives, ranging from $8 to $32, similar to the ‘other’ category ($10 to $40). Discretionary foods cost between $2 and $16 across households, with HH3 having the largest weekly cost and HH4 having the lowest (Table 11).

##### MedDiet

Fruit, grain foods, dairy foods, and meat and alternatives contributed similar amounts for the MedDiet baskets, ranging from $14 to $53, $12 to $43, $12 to $48 and $10 to $41, respectively. Vegetable cost contribution was slightly higher, ranging between $18 and $61 across households. The ‘other’ category had a cost ($9–$26) of around two times more than discretionary ($4–$13) (Table 11).

##### Western Diet

The highest cost contributor in the Western Diet basket was discretionary foods, ranging from $59 to $217 across households (Table 11). Fruit, grain foods, dairy foods, and the ‘other’ category had similar cost contributions, ranging from $3 to $16, $2to $15, $2 to $14, and $5 to $18, respectively. Vegetables had the lowest cost contribution ranging from $ < 1 to $15, with HH3 purchasing the higher amount of vegetables.

## 4. Discussion

### 4.1. Overview of Key Results

Food baskets and dietary modelling provide the opportunity to encourage healthy dietary patterns. Our research presents an updated food basket modelled on the Australian Guide to Healthy Eating and the first modification of a MedDiet for Australians. We further develop a Western Diet food basket not intended for use, but to act as a comparator, representing the current diet of Australians based on historical health surveys. Our results suggest that food baskets modelled on the AGHE and MedDiet have a superior nutrition profile compared to the dietary intake of a typical Australian. The Western Diet was significantly low in the five core food groups, and supplied up to six servings of energy dense discretionary foods per day. However, despite the higher number of discretionary servingss from the Western Diet, the MedDiet typically supplied the highest energy, ranging from 7634 kJ to 9859 kJ per day, likely due to the increased amount of energy and nutrient dense nuts, needs, and extra virgin olive oil compared to the AGHE. The AGHE basket met the food group recommendations and were mostly within the macronutrient distribution guidelines, except for carbohydrates, which were typically not within the recommended range (45–65% daily energy), and all NRVs were met. Similarly, the MedDiet met all NRVs except for Zinc (44M 98%), whereas all household members in the Western Diet were at inadequate intakes for at least three or more essential micronutrients including calcium, iron, zinc, vitamin A, fibre, and potassium.

Beyond the nutrition profile, we used a previous methodology to calculate the cost of the three food baskets. Generally, the MedDiet baskets were the most affordable, ranging from $78 to $285 across households compared to the AGHE ($75 to $315) and the Western Diet ($80 to $313), though all were relatively similar, with costs decreasing with a lower number of household members. The Western Diet required the highest initial outlay for HH1 ($504) compared to the AGHE ($481) and MedDiet ($467). The percentage of income was also relatively similar across baskets and households, with HH3 (elderly pensioners) requiring the highest proportion of income due to the pension providing less income per week ($744/week). This data indicates that following a MedDiet may not be a financial barrier as once thought due to the inclusion of higher cost products including good quality extra virgin olive oil, nuts, seeds, and fish.

### 4.2. Nutrition

Food modelling has previously been completed either over 7 [24,25,44] or 14 days [17,22,23,26,27]. In the current research, we opted to create meal plans across 7 days. Though our meal plans did not meet all initial aims, it is expected that over time nutrient and food group intake would probably level out and be considered adequate. For example, our AGHE-based meal plan failed to meet macronutrient distribution guidelines for carbohydrates (45–65% daily energy), ranging from 42 to 47% despite household members having sufficient servings per day of breads, cereals, and grain foods (6.1–9.0 servings per day) and adequate fruit and vegetable intake. The Victorian Healthy Food Basket (VHFB), which was based on the Queensland Healthy Food Basket methodology, had four reference families and met between 53 and 55% of daily energy from carbohydrate [27]. The VHFB basket included white bread, whole meal bread, crumpets, Weet-bix, instant oats, pasta, white rice, and biscuits. Our AGHE basket included similar products, including wholegrain bread, whole meal English muffins, brown rice, Weet-bix, rye wraps, and whole meal pasta, amongst others; however, it was only modelled across 7 days compared to the VHFB, which was across 14 days. Though the MedDiet basket only provided 37–39% of daily energy from carbohydrates, this is consistent with Mediterranean Diet guidelines (40% daily energy from carbohydrate) [37,38].

However, in terms of NRVs, our MedDiet basket provided only 98% of zinc requirements (44M) despite the high intake of seafood (7/week) and nuts (6/week), and barely met iron requirements for the 44F (101%). Similarly, the AGHE basket barely provided sufficient (101%) zinc intake for the 77M and iron for the 44F (101%), indicating issues with meeting NRV requirements over 7 days. However, as zinc requirements are based on the RDI, this is likely much more than people generally require. The slightly lower levels of zinc and iron observed in the MedDiet reflect lower meat and red meat intake across the 7-day period, with the main contributions coming from milk, fish, Weet-bix, seeds, and cheese for the MedDiet and beef mince, lamb, seeds, and yoghurt for the AGHE. Efforts were directed towards increasing zinc intake, namely adding zinc rich nuts and seeds to meals, i.e., pumpkin seeds as opposed to brazil nuts, swapping rolled oats for Weet-bix, swapping other fish for trout, and changing from couscous to quinoa, which contains around three times the zinc content. Other food modelling and healthy food basket research has detected issues with meeting zinc and iron for males and females, respectively. In the VHFB, the single adult household (adult male > 31 years old) only met 99% of zinc requirements [27]. Similarly, the Illawarra healthy food basket (IHFB) failed to meet zinc requirements for a 65-year-old female (94%) [45]. However, both the VHFB and IHFB had relatively high iron and zinc intake for the remaining household members despite containing a majority of processed breads, grains, and cereals. Though not specified, it is possible that these items could be fortified, hence the increased iron and zinc content; on the other hand, they could simply be the result of including far more animal products (bacon, chicken, sausages, beef) across the 14 days compared to our 7 days. Fortified products can provide substantial amounts of zinc and iron and are often used strategically in vegetarian diets to ensure adequate intakes alongside foods such as tofu [46]. As both the VHFB and IHFB have increased animal products included and processed breads and cereals, this could explain the exceeded sodium intake (117–188% and 99–166%, respectively) [27,45], and showed similarities to the Western Diet basket we created, which provided 121–205% of daily sodium recommendations. Both the AGHE and MedDiet baskets in the current research either met the recommendation (100%) for sodium or were below, with the MedDiet having slightly lower sodium intake across the 7 days. Decreasing sodium intake could be achieved by choosing sodium-reduced products, through swapping white wraps with rye, or by swapping processed meat such as salami with a leaner cut of leg ham.

Not all Mediterranean Diet food group recommendations were met (daily and weekly servings). To account for potential difficulty with dentition for the elderly pensioner household, weekly servings of nuts were <5 for the male and female (3.8 and 2.7 servings, respectively). Additionally, the 7-year-old male had a lower intake of nuts (3.9 servings per week) and seafood (1.4 serveings per week) to account for child preferences and potentially picky eaters. Further, these MedDiet guidelines are recommended for adults, as a Mediterranean Diet in children and adolescents is understudied compared to adult and older populations. However, research does suggest that MedDiet adherence is moderate to low in children and adolescents [47]. A general MedDiet pyramid for children and adolescents does not seem to exist, though there are separate guidelines for different countries, i.e., Greece [48] and Albania [49]. The Greek or Hellenic guidelines recommend 1–2 servings per day of vegetables; however, the serving is equal to 150–200 g (raw or cooked), whereas our Australian guidelines denote a serving of vegetables as approximately 75 g (half that of the Greek guidelines), meaning that the recommended number of servings is more likely to be 2–3 servings per day in an Australian context. Our MedDiet basket provided 5.1 servings of vegetables, which is approximately double that of the recommendations, which may be unfeasible for a child this age to consume.

Though our modelling may fall short in some circumstances, the Western Diet basket is illuminating in regard to typical Australian dietary intake. Long-term iron, zinc, calcium, potassium, fibre, and vitamin B6 deficiencies can result in chronic health conditions and associated complications, not only threatening to general health but quality of life.

### 4.3. Cost

Comparing the cost of the current food baskets to previous research is complex. We used different methodologies including a range of home-brand and more premium products in hopes of reflecting the true purchasing habits of consumers, and had different household structures in some circumstances. The 2014 Queensland Healthy Food Access Basket (QLD HFAB) cost $63, $117, $164, and $227 for a 1-, 2-, 3-, and 4-person household per week [22], respectively, while our AGHE and MedDiet meal plans ranged from $75 to 315 and $78 to $285, respectively. The costs are similar despite inflation and some differences within household structure, i.e., gender and no mention of the age of household members. Our research demonstrates a MedDiet can be just as if not more financially feasible in an Australian population than a basket based on our Australian guidelines and typical population intake. Due to this, MedDiet healthy food baskets could be offered as an alternative or healthy food baskets could be modified to mimic a MedDiet-styled pattern. Further, interestingly, the MedDiet food baskets provided the most nutritional value (energy) for an equivalent or lesser cost.

Additionally, we include far more non-core or ‘other’ foods including spreads, condiments, tea and coffee, and discretionary foods such as honey, sugar, ice-cream, and biscuits than other food baskets. The VHFB [27] only includes 3 non-core foods, margarine (polyunsaturated), white sugar, and canola oil, while the IHFB includes 13 with the additions of cake, soft drink, chocolate milk, coffee, milo, and other snack foods [45]. The Queensland Healthy Food Access Basket (QLD HFAB) includes no non-core foods and typically has the highest cost contribution from vegetables and legumes and lean meats and poultry across 7 days for different households ($17–$57 and $20–$67, respectively) [22], which is consistent with both our AGHE and MedDiet baskets.

Thoughthe inclusion of more non-core foods was utilised in hopes of better reflecting consumer purchasing trends and dietary intake, this may have impacted cost, making our baskets more expensive than others. Additionally, the proportion of home-brand to premium brands may be skewed in one direction in real life, thus skewing costs to be more affordable or more expensive. Though we decided not to include alcohol in either the MedDiet or AGHE basket, we acknowledge that both dietary patterns sanction the consumption in small amounts, which would increase weekly grocery costs.

Increasing the affordability of food baskets and dietary modelling can be approached from various angles. For example, using more home-brand products as opposed to the premium brands, purchasing frozen, canned, or tinned products as opposed to fresh, and bulk buying can be cost-saving mechanisms. Since the early development of food baskets in the 2000′s, there are many more home brand products available that often display similar labelling and compete against the branded products.

Research suggests that fruit and vegetable costs are more variable across time as opposed to meats, breads and cereals, dairy products, and non-core foods including oil and margarine [50]. The QLD HFAB demonstrated a price reduction of $21–$57 across a week for different households when only calculating the cost of the basket (49 items) using generic or home-brand products [22]. This is especially important when considering the role of food security and income. We compared the cost of the AGHE, MedDiet, and Western Diet food baskets to the average weekly income as a marker of food security, denoting that if baskets were >25% income there could be a risk of food security [9,16,51]. Between 5 and 21% of Australians may experience food insecurity, which refers to insufficient access to affordable and culturally appropriate food and beverages [15,16].

As income decreases, households have less disposable income that could be directed towards food and grocery shopping. Though all baskets were <25% of income based on the cost of the weekly meal plan (food eaten within the week), in the initial outlay, that is, purchasing multiple units if required (food lasting beyond the week), HH3 ranged from 34 to 42% across the AGHE, MedDiet, and Western Diet, indicating vulnerable populations or low-income earners may struggle to meet the initial outlay costs. Further, using the average weekly earnings of Australia may not truly reflect average weekly income due to differences within states and territories (weekly earnings) and employment status. Previous food baskets calculate assumed income based on average weekly income [24,25] or welfare payments and government assistance, meaning that the average weekly income for Australian households such as the ones used in this research may be earning far less, and therefore the proportion of income could be greater than anticipated. However, the QLD HFAB made assumptions such that the single-person household was unemployed and receiving government assistance of $306/week, with the basket requiring 20.3% of income [22], while our single-person household was based on a full-time worker earning $1835/week and the basket required between 4 and 11% (AGHE) and 4 and 12% (MedDiet) of income.

### 4.4. Limitations

Food modelling provides the opportunity to highlight the importance of personalized dietary advice and the complexity in meeting NRV requirements and satisfying government healthy eating guidelines such as the Australian Guide to Healthy Eating. Previous research has addressed concerns with generic meal plans that are not appropriate for the majority of the population and does not cater to individual needs [52], which can be detrimental to health. However, food modelling can be relatively subjective and impacted considerably depending on the chosen products. Though researchers aim to deter from bias, following pragmatic methodology, some decisions could be considered arbitrary.

Though the current research suggests tailored food baskets and meal plans based on the AGHE and MedDiet modified for Australians that can be nutritionally adequate over one week and cost similar amounts, there are various limitations with the current research. Firstly, the reference families selected are based on the average family composition characteristics of Australian families rather than a specific state. Though the purpose of the current research was not to create a state specific AGHE and MedDiet food basket, we acknowledge that differing household structures, i.e., multigenerational and shared households, may be more prevalent and more common in individual states. Additionally, no household members with food intolerances, food avoidance, allergies or ethical, cultural and or religious beliefs are included which may be a considerable portion of the Australian population [1]. Common foods that could be removed from dietary modelling include cow’s milk and dairy, gluten-containing foods and beverages, shellfish, peanuts, and pork [1]. Further, the predicted energy requirements (kJ/day) calculated may vary either in under or overestimation due to an individual’s height, weight, physical activity levels, and ethnicity, but they act as a reference point.

As the meal plans were developed to meet all the food group recommendations and NRVs, in some circumstances, an additional 2–4 servings above the recommendation were observed, i.e., 9 servings of grain for the 44-year-old male (AGHE basket) and 8.3 servings of vegetables for the 70-year-old female (MedDiet basket). Therefore, this could be an unrealistic amount of food for individuals to consume. Despite this, the quantities in the basket are similar to other baskets with similar household compositions.

When developing the Western Diet food basket which was modelled on information from the Australian Health Survey including reported energy intake (kJ/day), reported servings of food groups per day, and commonly consumed foods from each of the five food groups, discretionary included, there is the possibility the meal plan created does not truly reflect a Western Diet. Considering this style of survey is completed infrequently with the major sources of information from the 2011–2012 Australian Health Survey and limited available information and data cubes from the 2017–2018 Health Survey, accuracy may be reduced.

Additionally, we calculated the cost of the food baskets using one metro suburb in Adelaide, South Australia, using an online shopping tool (Coles, Australia online). The use of generic and branded products was intended; however, in some circumstances, the reduced fat, salt, or sugar product was only offered as a home-brand product. Previous food baskets have resorted to in-person surveys and gathered food prices from different socioeconomic areas. Though prior to gathering costs for the current research, we undertook a pilot to test differences in prices of staple products, i.e., bread, milk, and cereal across various Australian states with minimal difference. It is acknowledged that the price of the basket may differ to a small degree in other Australian states and may not truly be representative or comparable to previous work due to using an online supermarket tool despite increasing accessibility of gathering food prices. However, as mentioned, the focus of the current research was not to survey food costs in different areas or monitor affordability, but to present an updated AGHE food basket and present the first modified Mediterranean Diet food basket that aligns with Australian Dietary Guidelines. Using the average weekly earnings of Australian’s may also not be truly reflective and allow comparison with food insecurity data with individuals and family households either earning more or less than the national average. Further, this data exclude members of the defense force, employees of embassies, casual employees, self-employed persons, and employees paid under the Australian Governments Paid Parental Leave Scheme [53]. Previous food baskets have calculated family household income based on Centrelink payments, Newstart employment payments, childcare benefits and rebate, family tax (A and B), parenting payments, clean energy supplements, rent assistance [22], and other assumptions, while others are categorized based on welfare income, low income, and average income [44]. There is no standardized methodology for calculating income for reference families to our knowledge; however, access to weekly average income for Australians is updated relatively frequently through census surveys and routine data collection.

## 5. Conclusions

Food modelling has complexities but is useful to demonstrate the difficulties in meeting all nutritional requirements across a person’s lifespan. Minor changes in servinh sizes and food and beverage products can have profound impacts on the ability to satisfy dietary guidelines and recommendations. According to our research, both the AGHE and MedDiet basket have a more favourable nutrition profile and are generally cheaper compared to the Western Diet, which was modelled on a typical Australian’s intake and food preferences. Australians with consistently low intake of essential micronutrients, vitamins, and minerals may be at risk for long-term nutritional deficiency and associated complications.

### Future Directions

Future food basket research could consider a wider range of reference families including age and different ethnicities. Further, households could include individuals with allergies, aversions, or intolerances, or provide meal alternatives to increase reach, and consider including athletes or individuals with increased or decreased requirements. Food basket researchers could consider providing more detailed nutrition information and analyses including servings per day of food groups and energy contribution from macronutrients for comparative purposes.

Additionally, researchers could consider developing modified baskets including alcohol to demonstrate responsible drinking consistent with guidelines (<5% dietary energy) [54,55] across a 7- or 14-day period and the impact of alcohol consumption on increased energy intake. Over 25% of Australians aged 18 years and over exceeded the Australian Adult Alcohol guideline during 2020–2021, with men more likely (34%) to exceed than women (19%) [56]. Alcohol has been referred to as a part of Australian culture with its consumption occurring in various social circumstances, though typically consumed at levels too high (>10 standard drinks over 7 days) [56]. Conversely, the Mediterranean Diet promotes a conservative intake of red wine rich in resveratrol and consumed at social settings and gatherings [37]. Fortified products and cost-saving strategies could also be applied to increase nutritional adequacy while reducing overall cost.

Further, data from the current research could be used to apply cost-saving choices to the baskets to determine the cheapest basket possible, i.e., only generic brand, on-sale, canned, frozen, and bulk purchasing, which may be suitable for low-income earners. Mediterranean Diet food modelling should be further refined to apply to the Australian population, namely young children and adolescents, as the nutrition guidelines are unclear. Additionally, future food basket modelling should consider including more “other”, “non-core”, or “additional” foods such as tea, coffee, spreads, and condiments to more accurately reflect consumer purchasing habits, as it is somewhat unlikely individuals in households would only be consuming the foods and items within the basket. Baskets with limited assortment of core foods should also be reviewed considering there are recommendations around increasing dietary variety, namely aiming for 30 different biological food types per week. As the area of healthy food basket research progresses, the natural next step would be to implement these food baskets within a research trial. Research trials would allow statistical modelling and health outcomes to be assessed.

## Figures and Tables

**Table 1:** Household and family characteristics used for the healthy food basket analysis.

Abbreviation	Household Classification	Family Characteristics
HH1	Couple with children	44-year-old female 44-year-old male 7-year-old male 15-year-old female
HH2	Single parent with children	44-year-old female 7-year-old male 15-year-old female
HH3	Elderly pensioners	70-year-old female 70-year-old male
HH4	Single adult	30-year-old female

HH = household.

**Table 2:** Calculated income for the four reference households.

Abbreviation		Income (AUD$/Week)
HH1 ^a^	Couple with children	3670
HH2 ^a^	Single parent with children	1835
HH3 ^b^	Elderly pensioners	774
HH4 ^a^	Single adult	1835

^a^ Average weekly earnings refer to weekly gross income excluding salary sacrifice for an Australian adult working full time as calculated by the Australian Bureau of Statistics [31]. ^b^ Weekly adult pension rates (partnered) include base, supplement, and energy supplement as of September 2022 [32,33].

**Table 3:** Summary of energy requirements for all reference household family members, and in comparison to reported Australian dietary intake.

Household Member	AHS Reported Energy Intake (kJ)	Approximate Energy Intake Required (kJ)
70F	7270	8700
70M	9350	8700
44/30F	7540	8700
44M	1020	8700
15F	8110	9500
7M	7640	6200

Energy intake from 2011 to 2012 Australian Health Survey (24 h recall) [1].

**Table 4:** Overview of average daily food group and discretionary intake and energy contributions for the AGHE food basket.

AGHE											
	**Average Intake per Day**						**Average per Day**				
**Food Groups**	**Fruit**	**Veg**	**Grain**	**Dairy**	**Meat/Alt**	**Discr**	**En (kJ)**	**%enCHO**	**%en Fat**	**%EnSFA**	**%enPRO**
Recommended Servings	2	5	3–4.5	3.5 to 4	2	0–2.5	~8700	45 to 65	<30	>10	15 to 25
70F	2.7	5.7	7.1	3.7	2.4	1.5	8303	45	26	8	24
70M	2.4	5.7	6.9	4.1	3.2	2.5	9082	42	28	8	24
	**Average intake per day**						**Average per day**				
**Food Groups**	**Fruit**	**Veg**	**Grain**	**Dairy**	**Meat/alt**	**Discr**	**En (kJ)**	**%enCHO**	**%en Fat**	**%EnSFA**	**%enPRO**
Recommended Servings	2	5–6	6	2.5	2.5–3	0–3	~8700	45 to 65	<30	>10	15 to 25
44F	2.5	6.5	8.6	2.9	3.2	1.5	8723	45	28	8	22
44M	2.5	5.7	9.0	3.2	3.5	2.0	8967	44	27	7	23
	**Average intake per day**						**Average per day**				
**Food Groups**	**Fruit**	**Veg**	**Grain**	**Dairy**	**Meat/alt**	**Discr**	**En (kJ)**	**%enCHO**	**%en Fat**	**%EnSFA**	**%enPRO**
Recommended Servings	2	5	7	2.5	2.5	0–2.5	~9500	45 to 65	<30	>10	15 to 25
15F	2.7	5.2	8.3	3.9	3.0	1.5	8683 *	44	28	7	22
	**Average intake per day**						**Average per day**				
**Food Groups**	**Fruit**	**Veg**	**Grain**	**Dairy**	**Meat/alt**	**Discr**	**En (kJ)**	**%enCHO**	**%en Fat**	**%EnSFA**	**%enPRO**
Recommended Servings	1.5	4.5	4	2	1.5	0–2.5	~6200	45 to 65	<30	>10	15 to 25
7M	2.1	5.6	6.1	2.3	2.1	1.7	6529 *	47	25	7	22

* room for additional discretionary sources; Disc = discretionary foods; M = male; F = female; kJ = kilojoules; En = energy; CHO = carbohydrate; SFA = saturated fatty acid; PRO = protein; 70F = year-old female, 70M = 70-year-old male; 44F = 44-year-old female; 44M = 44-year-old male; 15F = 15-year-old female; 7M = 7 year old male.

**Table 5:** Overview of average daily and weekly food group and discretionary intake and energy contributions for the MedDiet food basket.

MedDiet															
	Per Day					Per Week					Energy and Macronutrients				
Food Groups	Fruit	Veg	Grain	Dairy	EVOO	Red Meat	Seafood	Legumes	Nuts	Disc	En (kJ)	%en CHO	%en Fat	%En SFA	%en PRO
Recommended Servings	2–3	≥5	4–6	2	3	1	3	3	5	0–2	6500–9500	40	40	<10	20
70F	2.1	8.3	6.0	3.1	3	0.7	4.8	5.4	2.7	1.5	9057	37	37	8	20
70M	2.2	7.9	6.6	2.9	3	0.7	6.2	5.5	3.8	1.5	9297	38	38	8	20
44F	2.9	7.5	7.6	2.4	3	1.0	3.1	5.2	4.7	0.5	9450	39	39	8	17
44M	2.1	7.0	8.5	2.1	3	1.1	7.4	5.5	6.0	0.5	9859	38	38	8	19
15F	2.2	6.0	7.8	3.3	3	1.1	3.2	3.0	5.5	1.3	9556	38	38	8	19
7M	2.0	5.1	5.8	2.7	2.5	0.8	1.4	3.6	3.9	1	7634	38	38	9	18

M = male; F = female; 70F = 70-year-old female; 70M = 70-year-old male; 44F = 44-year-old female; 44M = 44-year-old male; 15F = 15-year-old female; 7M = 7-year-old male. EVOO = extra virgin olive oil; Disc = discretionary foods; kJ = kilojoules; En = energy; CHO = carbohydrate; SFA = saturated fatty acid; PRO = protein; 6500 kJ refers to approximate energy intake for a 7-year-old child and 9500 kJ for the 15-year-old female, while 8700 kJ is the approximate energy intake required for an adult.

**Table 6:** Overview of average daily food group and discretionary intake and energy contributions for the Western Diet food basket.

Western Diet											
	**Per day**						**Energy**				
**Food Groups**	**Fruit**	**Veg**	**Grain**	**Dairy**	**Meat/alt**	**Discr**	**En (kJ)**	**%enCHO**	**%en Fat**	**%EnSFA**	**%enPRO**
Recommended Servings	2	5	3–4.5	3.5 to 4	2	0–2.5	~8700	45–65	<30	>10	15 to 25
Reported intake	1.4	3.1	4.2	1.2	1.9	5.7	7300–9400	43	32	12	18
70F	1.4	3.3	4.3	1.2	1.8	5.6	8320	43	40	17	14
70M	1.5	3.4	4.5	1.5	1.8	6.0	9456	43	40	17	14
	**Per day**						**Energy**				
**Food Groups**	**Fruit**	**Veg**	**Grain**	**Dairy**	**Meat/alt**	**Disc**	**En (kJ)**	**%enCHO**	**%en Fat**	**%EnSFA**	**%enPRO**
Recommended Servings	2	5–6	6	2.5	2.5–3	0–2.8	~8700	45–65	<30	>10	15 to 25
Reported intake	1.2	2.8	4.5	1.5	1.9	6.2	7540–10,220	44	33	12	18
44F	1.2	2.7	4.9	1.6	1.9	5.7	8780	44	39	14	15
44M	1.1	2.9	5.2	1.9	2.2	5.9	9958	48	35	14	14
	**Per day**						**Energy**				
**Food Groups**	**Fruit**	**Veg**	**Grain**	**Dairy**	**Meat/alt**	**Disc**	**En (kJ)**	**%enCHO**	**%en Fat**	**%EnSFA**	**%enPRO**
Recommended Servings	2	5	7	2.5	2.5	0–3.2	~9500	45–65	<30	>10	15 to 25
Reported intake	1.5	1.9	4.6	1.5	1.3	7.0	8110	49	34	14	16
15F	1.5	1.9	4.3	1.6	1.3	6.0	8450	48	36	17	13
	**Per day**						**Energy**				
**Food Groups**	**Fruit**	**Veg**	**Grain**	**Dairy**	**Meat/alt**	**Disc**	**En (kJ)**	**%enCHO**	**%en Fat**	**%EnSFA**	**%enPRO**
Recommended Servings	1.5	4.5	4	2	1.5	0–2	~6200	45–65	<30	>10	15 to 25
Reported intake	1.6	1.4	3.6	1.6	0.8	5.9	7640	52	32	14	15
7M	1.6	1.4	3.6	1.8	1.0	5.9	7800	46	40	20	12

70F = 70-year-old female; 70M = 70-year-old male; 44F = 44-year-old female; 44M = 44-year-old male; 15F = 15-year-old female; 7M = 7-year-old male; Disc= discretionary foods; M = male; F = female; kJ = kilojoules; En = energy; CHO = carbohydrate; SFA = saturated fatty acid; PRO = protein.

**Table 7:** Summary of average daily nutrient intake by household member for the AGHE food basket.

AGHE	Household Member					
Dietary Component	77F	77M	44F	44M	15F	7M
Energy (kJ)	8303	9082	8724	8967	8683	6529
PRO (%en)	24	24	22	23	22	22
Fat (%en)	26	28	28	27	28	25
Sat fat (%en)	8	8	8	7	8	7
P:M:S	20:46:34	24:32:44	24:45:31	26:45:29	27:44:30	25:42:33
CHO (%en)	45	42	43	44	44	47
Vitamins and minerals (% NRV)						
Fibre ^a^	143	126	188	151	210	193
Thiamin	159	161	132	151	135	233
Riboflavin	254	242	208	210	247	461
Niacin	330	327	361	336	322	461
Vitamin C	613	648	420	497	476	389
Vitamin E ^a^	259	230	207	192	193	194
Vitamin B6	157	144	162	158	155	246
Vitamin B12	206	210	198	186	202	371
Folate, DFE	242	231	208	220	203	450
Vitamin A	228	191	293	293	222	329
Sodium ^b^	100	100	96	99	98	100
Potassium ^a^	168	134	172	130	179	145
Magnesium	154	140	223	169	191	353
Calcium	115	166	154	148	128	151
Phosphorus	204	234	228	238	194	332
Iron	155	178	101	203	110	132
Zinc	155	101	198	110	215	296
Selenium	158	150	118	111	172	184
Iodine	146	139	130	114	148	232
Linoleic Acid ^a^	101	100	160	101	178	102
Alpha-Linolenic Acid ^a^	195	111	161	105	114	122
VLCN3 ^b^	116	102	113	100	115	73

^a^ = Adequate Intake; ^b^ = Suggested Dietary Intake, VLCN3 (EPA, DPA, DHA) refers to SDT (min) sodium refers to SDT (max) for adults and Upper Level (UL) of intake for children; Only an Adequate Intake is available for VLCN3 for a 7 year old; 70F = 70-year-old female; 70M = 70-year-old male; 44F = 44-year-old female; 44M = 44-year-old male; 15F = 15-year-old female; 7M = 7-year-old male.

**Table 8:** Summary of average daily nutrient intake by household member for the Mediterranean food basket.

MedDiet	Household Member					
Dietary Component	77F	77M	44F	44M	15F	7M
Energy (kJ)	9057	9297	9451	9859	9556	7635
PRO (%en)	20	20	17	19	19	18
Fat (%en)	37	38	39	38	38	38
Sat fat (%en)	8	8	8	8	9	9
P:M:S	16:60:24	19:58:23	18:60:22	20:58:22	18:57:24	17:58:25
CHO (%en)	37	38	39	38	38	38
Vitamins and minerals (% NRV)						
Fibre ^a^	184	149	197	178	209	190
Thiamin	135	172	173	140	140	193
Riboflavin	235	178	195	168	240	308
Niacin	328	301	304	285	306	381
Vitamin C	575	595	526	655	588	538
Vitamin E ^a^	344	270	346	270	278	267
Vitamin B6	130	124	154	155	151	231
Vitamin B12	282	331	191	244	247	387
Folate, DFE	212	218	260	225	195	333
Vitamin A	245	202	415	219	311	319
Sodium ^b^	100	87	93	98	88	86
Potassium ^a^	149	113	160	119	168	158
Magnesium	133	130	185	146	159	337
Calcium	120	131	144	167	111	180
Phosphorus	203	232	189	215	177	337
Iron	176	222	101	221	103	110
Zinc	152	104	162	98	195	257
Selenium	240	158	133	198	147	184
Iodine	142	126	132	135	130	234
Linoleic Acid ^a^	137	102	182	121	184	138
Alpha-Linolenic Acid ^a^	123	104	162	105	153	107
VLCN3 ^a,b^	199	247	121	207	125	596

^a^ = Adequate Intake; ^b^ = Suggested Dietary Intake, VLCN3 (EPA, DPA, DHA) refers to SDT (min) sodium refers to SDT (max) for adults and Upper Level (UL) of intake for children; Only an Adequate Intake is available for VLCN3 for a 7 year old; 70F = 70-year-old female; 70M = 70-year-old male; 44F = 44-year-old female; 44M = 44-year-old male; 15F = 15-year-old female; 7M = 7-year-old male.

**Table 9:** Summary of average daily nutrient intake by household member for the Western Diet food basket.

	Household Member					
Dietary Component	77F	77M	44F	44M	15F	7M
Energy (kJ)	8320	9460	8790	9957	8450	7840
PRO (%en)	14	14	15	14	13	12
Fat (%en)	40	40	39	35	36	40
Sat fat (%en)	17	17	14	14	17	20
P:M:S	13:42:45	13:41:46	16:45:40	15:43:42	13:36:51	11:34:55
CHO (%en)	43	43	44	48	48	46
Vitamins and minerals (% NRV)						
Fibre ^a^	74	72	54	52	46	56
Thiamin	127	165	100	86	101	161
Riboflavin	121	135	126	114	115	217
Niacin	205	237	270	221	218	303
Vitamin C	321	121	363	337	376	409
Vitamin E ^a^	128	107	166	108	82	85
Vitamin B6	54	59	67	78	77	140
Vitamin B12	174	183	153	176	122	268
Folate, DFE	143	163	135	108	121	199
Vitamin A	142	78	68	65	67	126
Sodium ^b^	179	205	145	141	121	162
Potassium ^a^	91	63	90	62	81	88
Magnesium	73	51	73	56	59	153
Calcium	51	68	69	76	48	95
Phosphorus	134	156	113	127	88	207
Iron	105	119	38	96	49	61
Zinc	111	72	113	75	120	174
Selenium	97	92	93	82	86	134
Iodine	111	115	102	107	95	154
Linoleic Acid ^a^	107	80	129	77	100	90
Alpha-Linolenic Acid ^a^	242	162	355	197	179	130
VLCN3 ^b^	25	19	26	17	19	177

^a^ = Adequate Intake; ^b^ = Suggested Dietary Intake, VLCN3 (EPA, DPA, DHA) refers to SDT (min) sodium refers to SDT (max) for adults and Upper Level (UL) of intake for children; Only an Adequate Intake is available for VLCN3 for a 7 year old; 70F = 70-year-old female; 70M= 70-year-old male; 44F = 44-year-old female; 44M = 44-year-old male; 15F = 15-year-old female; 7M = 7-year-old male.

**Table 10:** Summary of AGHE, MedDiet, and Western Diet baskets per household including the weekly cost and initial outlay, and in relation to percentage of average income.

**AGHE**	**HH1**	**HH2**	**HH3**	**HH4**
Cost of weekly meal plan ($)	315	238	186	75
Cost of initial outlay ($)	481	388	314	204
Income (%)	9–13	13–21	24–42	4–11
**MedDiet**	**HH1**	**HH2**	**HH3**	**HH4**
Cost of weekly meal plan ($)	285	211	135	78
Cost of initial outlay ($)	467	365	265	220
Income (%)	8–13	11–20	17–34	4–12
**Western Diet**	**HH1**	**HH2**	**HH3**	**HH4**
Cost of weekly meal plan ($)	313	217	157	80
Cost of initial outlay	504	385	302	217
Income (%)	9–14	12–21	20–40	4–12

HH = household; HH1 = couple with two kids; HH2 = single parent with two kids; HH3 = elderly pensioners; HH4 = single adult; percentage income based on an Australian average weekly income of AUD$3670 for HH1, AUD$1835 for HH2, AUD$774 for HH3, and AUD$1835 for HH4.

**Table 11:** Cost contribution of weekly food basket meal plan, not initial outlay, per AGHE food group.

Basket	Food Group/Category ($)						
**AGHE**	**Fruit**	**Vegetables**	**Grain**	**Dairy**	**Meat, Alt**	**Other**	**Disc**
HH1	32	70	44	45	74	40	10
HH2	25	57	30	32	52	36	6
HH3	27	40	21	32	30	21	16
HH4	8	19	6	11	21	10	2
**Med Diet**	**Fruit**	**Vegetables**	**Grain**	**Dairy**	**Meat, alt**	**Other**	**Disc**
HH1	53	61	43	48	41	26	13
HH2	39	44	37	39	23	20	10
HH3	16	33	18	26	25	13	3
HH4	14	18	12	12	10	9	4
**Western Diet**	**Fruit**	**Vegetables**	**Grain**	**Dairy**	**Meat, alt**	**Other**	**Disc**
HH1	16	5	15	14	27	18	217
HH2	14	4	8	10	17	13	150
HH3	6	15	6	9	12	16	94
HH4	3	<1	2	2	8	5	59

HH = household; other = oils, spreads, tea, coffee; Discretionary = biscuits, processed meats, honey, high fat spreads.

## Data Availability

Data is available when requested from the corresponding author.

## Supplementary Materials

The following supporting information can be downloaded at: https://www.mdpi.com/article/10.3390/nu15071692/s1, Table S1: Summary of daily energy requirements (~9500 kJ) for a 15-year-old for the HFB from the five food groups; Table S2: Summary of daily energy requirements (~6200 kJ) for a 7-year-old for the HFB from the five food groups; Table S3: Grocery list of foods and quantities in AGHE HFB; Table S4: Grocery list of foods and quantities in MedDiet HFB; Table S5: Grocery list of foods and quantities in Western Diet FB; Table S6: Example meals for AGHE HFB; Table S7: Example meals for MedDiet HFB; Table S8: Example meals for Western Diet food basket.
