# Supplementary material for: Developing a Mediterranean Healthy Food Basket and an Updated Australian Healthy Food Basket Modelled on the Australian Guide to Healthy Eating
Source: Nutrients. 2023 Mar 30;15(7):1692. doi: 10.3390/nu15071692 (PMC10096976; doi:10.3390/nu15071692)
Supplement: Supplementary file 1 [file nutrients-15-01692-s001.zip › nutrients-2294802-supplementary.pdf]

Supplementary Information

Table S1. Summary of daily energy requirements (~9,500kJ) for a 15-year-old for the HFB from the five food groups

15-year-old	Fruit	Vegetables	Grains	Meat	Dairy
Recommended Serves	2	5	7	2.5	3.5
Energy (kJ)	700	1750	3500	1500	2100

Table S2. Summary of daily energy requirements (~6,200kJ) for a 7-year-old for the HFB from the five food groups

7-year-old	Fruit	Vegetables	Grains	Meat	Dairy
Recommended Serves	1.5	4.5	4	1.5	2
Energy (kJ)	525	1575	2000	900	1200

Table S3. Grocery list of foods and quantities in AGHE HFB

**Food/Beverage item**	**HH1**	**HH2**	**HH3**	**HH4**
**Fruit**				
Apple	1148g	1148g	906g	-
Banana	2260g	1770g	1332g	777g
Orange Juice	2383ml	1439ml	684.6g	473ml
Mandarin	1725g	825g	525g	375g
Orange	810g	810g	-	324g
Kiwifruit	-	-	780g	-
**Vegetables and Legumes**				
Avocado	126g	126g	25g	25g
Broccoli/Broccolini	2170g	1747g	2323g	423g
Carrot	1068g	603g	775g	243g
Corn	71g	57g	-	28g
Cucumber	1715g	1015g	181g	181g
Spinach (frozen)	1398g	808g	80g	384g
Spinach (fresh)	426g	426g	137g	330g
Mixed lettuce	1033g	752g	297g	193g
Mixed vegetables (frozen)	900g	900g	400g	400g
Onion	423g	302g	289g	89g

Potato	355g	219g	921g	83g
Rocket	144g	86g	-	-
Tomato	1488g	998g	422g	358g
Green beans	-	-	94g	-
Bok choy	-	-	640g	-
Passata	-	-	188g	-
Tinned tomatoes	-	-	464g	-
**Breads and cereals (grain foods)**				
Wholegrain biscuit/cracker	113g	113g	34g	-
Wholegrain bread roll	210g	210g	70g	70g
Wholegrain bread	1162g	904g	1114g	324g
Wholemeal bread	-	-	160g	-
Whole meal pita bread	141g	141g	-	-
Breakfast cereal (grain, wheat, whole meal)	225g	-	98g	-
Couscous	276g	197g	79g	79g
Whole meal English muffin	67g	67g	67g	-
Whole meal pasta	930g	543g	295g	310g
Multigrain rice cake	1174g	774g	-	89g
Noodles, hokkien	-	-	255g	-
Rice cake	-	-	406g	-
Brown rice	628g	377g	530g	168g
Quinoa	-	-	96g	-
Rolled oats	680g	680g	144g	-
Weet-bix	260g	260g	72g	-
Wholegrain wrap	71g	71g	-	-
Rye wrap	141g	71g	142g	-
Bar from puffed rice	132g	132g	-	-
Bar fruit and nut	256g	256g	128g	192g
**Lean meats, poultry, fish, eggs, nuts, seeds**				
Red kidney beans	855g	617g	333g	285g
Chickpeas	86g	86g	-	86g
Chicken breast	615g	389g	446g	113g
Lamb chops	521g	337g	-	199g
Beef strips	-	-	275g	-
Ham (reduced salt)	-	-	275g	-
Eggs	468g	384g	154g	84g
Salmon	245g	165g	-	65g
Tuna	490g	350g	523g	70g
White fish	-	-	430g	-
Smoked salmon	150g	110g	-	50g
Beef mince	665g	470g	140g	250g
Turkey (reduced fat/salt)	120g	40g	-	-

Almonds	181g	153g	-	45g
Brazil nuts	46g	40g	-	13g
Walnuts	8g	8g	-	-
Pumpkin seeds	452g	331g	65g	131g
Sunflower seeds	-	-	133g	-
**Milk, yoghurt and cheese**				
Fetta (reduced fat)	110g	95g	-	55g
Cream cheese (reduced fat)	20g	20g	-	20g
Mozzarella cheese (reduced fat)	193g	139g	40g	35g
Cheddar cheese (reduced fat)	-	-	210g	-
Cow’s Milk (reduced fat)	9962ml	8178ml	3933ml	2111ml
Yoghurt (reduced fat)	5070g	3250g	4615g	1387g
**Fats and oils**				
Olive oil	487ml	365ml	400ml	123ml
Monounsaturated spread (reduced fat)	62g	25g	109g	10g
**Other**				
Coffee powder	22g	-	45g	-
Dip, eggplant	100g	100g	-	-
Dip, hummus	165g	80g	-	-
Dressing, French, fat free	170ml	128ml	-	50ml
Chocolate drinking powder, (reduced fat/sugar)	155g	155g	-	-
Honey	36g	36g	58g	-
Vegemite spread	5g	3g	10g	-
Ready meal, Lasagna	600g	400g	-	150g
Peanut butter (reduced fat/salt)	15g	15g	40g	10g
Strawberry jam	-	-	56g	-
Popcorn (reduced fat/salt)	310g	220g	-	-
Sauce, chili	56g	56g	20g	28g
Sauce, salsa	198g	176g	-	88g
Sauce, honey soy	-	-	136g	-
Sauce, chutney	-	-	7g	-
Sauce, garlic aioli	-	-	20g	-
Teabags	13	13	-	13
Balsamic vinegar	56ml	42ml	-	14ml
Biscuit, sweet	-	-	130g	-

Table S4. Grocery list of foods and quantities in MedDiet HFB

Food/Beverage item	HH1	HH2	HH3	HH4
**Fruit**				

Apple	376g	376g	435g	-
Banana	2207g	1887g	1443g	888g
Orange	1620g	648g	-	-
Orange Juice	1943mL	1628mL	1155mL	840mL
Kiwifruit	78g	78g	-	78g
Mandarin	225g	225g	-	75g
Mixed dried fruit	35g	35g	-	35g
Mixed berries (frozen)	2712g	1782g	-	426g
Grapes	-	-	170g	-
**Vegetables and Legumes**				
Asparagus	238g	143g	-	-
Spinach (frozen)	1648g	1581g	181g	1016g
Spinach (fresh)	618g	303g	-	113g
Broccoli	1346g	876g	1980g	240g
Capsicum, red	248g	206g	233g	142g
Carrot	418g	271g	494g	206g
Celery	-	-	27g	-
Cauliflower	513g	386g	-	162g
Cucumber	276g	158g	196g	20g
Eggplant	100g	100g	100g	100g
Mixed lettuce	403g	368g	175g	70g
Mixed vegetable (frozen)	180g	180g	920g	-
Mushroom	415g	335g	173g	150g
Onion	482g	407g	367g	223g
Potato, white	285g	235g	-	115g
Potato, sweet	423g	172g	130g	84g
Tomato, fresh	1456g	719g	367g	244g
Passata	954g	767g	725g	467g
Zucchini	280g	234g	144g	167g
**Breads and cereals (grain foods)**				
Barley	49g	49g	-	49g
Bread, whole meal	244g	178g		56g
Bread, mixed grain	384g	144g	396g	144g
Bread, pita	528g	462g	56g	462g
Bread, rye & grain	504g	364g	615g	105g
Bread, sourdough	660g	512g	90g	-
Wrap, mixed grain	199g	199g	-	64g
Pasta, whole meal	543g	388g	581g	155g
Quinoa	135g	135g	141g	135g
Rice cake, multigrain	580g	330g	-	30g
Rice, brown	514g	389g	-	-
Rolled oats	165g	165g	-	-

Lasagne sheets	42g	42g	42g	42g
Weet-bix	1023g	621g	624g	291g
**Lean meats, poultry, fish, eggs, nuts, seeds**				
Bean, butter bean	180g	-	245g	-
Bean, chickpea	173g	173g	259g	173g
Bean, lentil, brown	361g	361g	68g	185g
Bean, mixed	-	-	190g	-
Chicken breast	50g	50g	230g	-
Chicken mince	100g	100g	-	100g
Beef mince	240g	-	60g	-
Eggs	664g	488g	452g	167g
Fish, salmon	268g	150g	-	75g
Fish, sardine	225g	-	870g	-
Fish, tuna	770g	350g	140g	140g
Fish, trout	-	-	80g	-
Fish, white	145g	145g	65g	65g
Nut, brazil	17g	-	53g	-
Nut, walnut	90g	78g	-	30g
Nut, mixed	101g	50g	15g	-
Seed, pumpkin	330g	255g	80g	75g
Sunflower seed	-	-	20g	-
**Milk, yoghurt and cheese**				
Cheese, cheddar (reduced fat)	115g	75g	-	-
Cheese, fetta (reduced fat)	70g	50g	97g	20g
Cheese, mozzarella (reduced fat)	185g	185g	150g	30g
Cheese, parmesan (reduced fat)	5g	5g	5g	5g
Cheese, ricotta (reduced fat)	280g	280g	-	200g
Cheese, Swiss	-	-	55g	-
Cow’s Milk (reduced fat)	10690mL	8512mL	6252mL	2393mL
Yoghurt, Greek	3520g	2680g	1810g	840g
**Fats and oils**				
Extra Virgin Olive Oil	1116mL	863mL	615mL	383mL
**Other**				
Biscuit, sweet	16g	16g	36g	-
Chocolate Drinking powder, (reduced fat/sugar)	29g	29g	-	-
Ham (reduced salt)	60g	60g	20g	60g
Honey	41g	41g	108g	12g
Mayonnaise (reduced fat)	28g	28g	28g	-
Peanut butter (reduced fat/salt)	40g	30g	20g	-
Popcorn (reduced fat/salt)	90g	90g	-	-
Turkey	280g	185g	-	95g
Dip, eggplant	60g	40g	-	40g

Dip, hummus	140g	60g	40g	20g
Balsamic vinegar	-	-	42g	-
Tea bags	9	9	29	9
Coconut milk	102mL	102mL	-	102mL

Table S5. Grocery list of foods and quantities in Western Diet FB

Food/Beverage item	HH1	HH2	HH3	HH4
**Fruit**				
Apple	-	-	593g	-
Banana	838g	838g	111g	-
Orange	1620g	1134g	-	1134g
Orange juice	5824mL	4524mL	1603mL	1040mL
Fruit salad, canned	440g	100g	408g	100g
**Vegetables and Legumes**				
Beans, green, frozen	-	-	140g	-
Broccoli, frozen	-	-	90g	-
Brussel sprouts	-	-	-	-
Carrot	-	-	83g	-
Cucumber	-	-	80g	-
Lettuce	-	-	53g	-
Mixed vegetables, frozen	43g	43g	326g	43g
Onion	12g	-	24g	-
Peas	125g	125g	-	-
Potato	421g	380g	279g	-
Snow peas, frozen	-	-	255g	-
Tomato	391g	331g	64g	-
**Breads and cereals (grain foods)**				
White bread	206g	137g	824g	396g
White bread roll	1511g	1207g	276g	69g
Breakfast cereal, corn	69g	28g	-	28g
Breakfast cereal, rice, cocoa	140g	140g	-	-
Rolled oats	-	-	60g	-
Pasta, white	-	-	581g	-
Snack/Muesli bar (no fruit)	186g	-	-	-
Snack/Muesli bar (chocolate)	312g	312g	17g	68g
Weet-bix	90g	90g	-	-
**Lean meats, poultry, fish, eggs, nuts, seeds**				
Beans, mixed, canned	-	-	380g	-
Beef strips	400g	240g	-	130g

Chicken breast	311g	198g	-	85g
Beef mince	339g	239g	350g	-
Sausages, beef (regular fat)	683g	481g	505g	202g
Mixed nuts	92g	20g	86g	20g
**Milk, yoghurt, and cheese**				
Cheese, cheddar (regular fat)	399g	294g	252g	63g
Cheese, parmesan (regular fat)	-	-	80g	-
Cow’s milk (regular fat)	4512mL	30000mL	1241mL	876mL
**Fats and oils**				
Butter/margarine spread (regular fat/salt)	264g	197g	176g	76g
Canola oil	487g	365g	324g	163g
**Other**				
Coffee powder	11g	-	21	-
Gravy powder	77g	58g	60g	19g
Vegemite	20g	15g	27g	5g
Peanut butter	112g	78g	-	68g
Tomato sauce	291g	270g	-	-
Tea bags	7	7	12	7
Chocolate bar	175g	175g	-	-
Biscuit, sweet	63g	56g	72g	56g
Biscuit, Anzac	-	-	221g	-
Biscuit, wafer style	-	-	14g	-
Cake	485g	485g	-	-
Chicken Kiev (frozen)	471g	351g	438g	120g
Chicken nuggets (frozen)	320g	320g	-	-
Chips, potato, packet	380g	320g	200g	240g
Chips, potato (frozen)	1020g	720g	410g	240g
Chocolate milk/Iced coffee	120mL	120mL	250mL	-
Beef, corned, sliced	390g	286	358	-
Jelly/jube lollies	379g	181g	23g	-
Drinking chocolate powder	12g	12g	-	-
Frozen meal (beef pasta)	300g	-	1583g	-
Fish cake, frozen	-	-	350g	-
Garlic aioli	-	-	50g	-
Honey	40g	40g	57g	-
Ice cream, caramel	660	660mL	-	-
Ice cream, coffee	792mL	-	528mL	-
Ice cream, cookies and cream	396mL	396mL	99mL	-
Ice cream, vanilla choc chip	660mL	660mL	396mL	660mL
Jam, strawberry flavoured	38g	38g	90g	8g
Muffin, berry flavoured	1640	980g	-	815
Pie, meat and cheese	568g	394g	138g	175g

Pikelets	125g	125g	-	-
Pizza, chicken, and bacon	106g	106g	-	-
Pizza, ham, and cheese	500g	300g	-	300g
Pizza, meat lovers	531g	319g	-	213g
Pudding, sticky date	-	-	1080g	-
Salami	288g	219g	-	69g
Sauce, chocolate	308g	308g	-	-
Sauce, pasta cream based	-	-	1060g	-
Sausage roll	910	650	-	260g
Soft drink, coca cola	3358mL	1408mL	-	-
Soft drink, lemonade	455mL	-	1500mL	-
Sugar, white	59g	29g	-	29g
Syrup, maple flavoured	10mL	10mL	-	-

Table S6. Example meals for AGHE HFB

	Breakfasts	Lunches	Dinners	Snacks
70F	• 0.5c rolled oats, 1c reduced fat milk, pinch cinnamon, 1 banana and 1tsp honey<br>• 2x slices grain bread, 1c spinach, 2 eggs, 2tsp reduced fat monounsaturated spread<br>• 2x slices grain bread, 20g peanut butter, 1 banana 2tsp reduced fat monounsaturated spread	• 2x slices grain bread with salt 50g ham, 21g reduced fat cheese with salad items (onion, lettuce, tomato), 1tsp chutney<br>• 1 grain or rye wrap with 50g roast chicken breast, 1c lettuce and salad, 2tsp garlic aioli<br>• 75g tin tuna, 4x whole meal crispbreads, 25g onion, 30g tomato<br>• 1 medium potato (~230g), 0.75c kidney beans, 0.5c tomato paste, 0.75c broccolini (steamed), 2tsp reduced fat monounsaturated spread, 20g reduced fat mozzarella	• 75g fish with 0.75c potato, 1c broccoli and 0.75c carrot<br>• 1c pasta, 75g beef mince, ¼ onion, 0.75c passata, 10g reduced fat mozzarella<br>• 1c brown rice, 65g stir fried beef strips, 0.5c bok choy, 0.6c broccoli<br>• 113g chicken breast fried in oil, 0.25c carrot (steamed), 0.75c broccoli (fried with chicken breast), 0.25c quinoa cooked in water, 1tb chili sauce	• 1.5c reduced fat yoghurt with apple<br>• ½ whole meal English muffin with ½tbsp strawberry jam<br>• Coffee/tea with yoyos<br>• 1.25c yoghurt with 10g sunflower seeds
70M	• 0.75c rolled oats, 1c reduced fat milk, pinch cinnamon, 1 banana and 1tsp honey, 15g pumpkin seeds<br>• 2x slices grain bread, 1c spinach, 2 eggs, 2tsp	• Grain bread roll (~70g), 50g reduced salt ham, 25g onion, 1.5c lettuce/spinach, 31g tomato, 21g reduced fat cheese<br>• 1 grain or rye wrap with 50g reduced salt ham, 1c lettuce and salad, 2tsp garlic aioli	• 75g fish with 0.75c potato, 1c broccoli and 0.75c carrot<br>• 1c pasta, 75g beef mince, ¼ onion, 0.75c passata, 10g reduced fat mozzarella<br>• 0.6c brown rice, 75g stir fried beef strips, 0.5c bok choy, 0.5c broccoli	• 1.25c yoghurt with 15g sunflower seeds, 5g sunflower seeds<br>• ½ whole meal English muffin with ½tbsp strawberry jam, kiwifruit<br>• Coffee/tea with yoyos

	reduced fat monounsaturated spread<br>• 0.75c bran flake/wheat bran cereal, 1c milk, pinch cinnamon, 1 banana, drizzle honey, 20g sunflower seeds	• 75g tin tuna, 4x whole meal crispbreads, 25g onion, 30g tomato	• 0.75c hokkien noodles, 0.5c carrot, 0.5c bok choy, 85g chicken breast, 0.25c honey soy sauce	
44F	• 0.75c rolled oats, 1c reduced fat milk, pinch cinnamon, 1 banana, 15g pumpkin seeds<br>• 80g weet-bix, 1c reduced fat milk, pinch cinnamon, 1 banana, 18g pumpkin seeds<br>• 2x slices grain bread, 1tsp reduced fat monounsaturated spread, 120g tomato, 50g spinach with a banana	• 2x slice grain bread, 15g reduced fat mozzarella, 1tsp reduced fat monounsaturated spread, 1.5c salad (lettuce, spinach, onion)<br>• Mixed grain bread roll (~70g), 2cups salad (lettuce, spinach, onion), 2tsp reduced fat monounsaturated spread<br>• 2x slice grain bread, 50g reduced salt ham, 50g spinach, 60g tomato, 1tsp reduced fat monounsaturated spread, 25g onion<br>• 50g smoked salmon, 2x slices grain bread, 25g avocado, 2x eggs, 1.5c baby spinach fried	• 65g fish with 0.5c brown rice, 1c broccoli and 0.75c carrot, 1tb chili sauce<br>• 1c whole meal pasta, 65g beef mince, 0.25c kidney beans, 0.75c passata, 10g reduced fat mozzarella, ½ small carrot, 50g spinach<br>• 0.25c couscous cooked in water, 0.75c broccoli, 27g carrot, 50g spinach fried in oil with 85g chicken breast, 45g reduced fat fetta crumbled on top<br>• 60g beef mince, 0.5c kidney beans, 1tbsp corn kernels, 0.25c brown rice, 70g spinach, 2tb salsa sauce	• 0.6c yoghurt, 15g almonds<br>• Tea/coffee with fruit and nut bar<br>• 70g tuna, 2 whole meal corn thins/crispbreads<br>• Tea/coffee with mandarin and fruit and nut bar<br>• Tea/coffee with 4x whole meal crispbreads/biscuits, 40g cucumber, 20g cream cheese reduced fat

44M	• 2x slice mixed grain bread, 5g vegemite, 1tsp reduced fat monounsaturated spread, 200ml orange juice<br>• 75g breakfast cereal (grain, rice, oat), 1.5c reduced fat milk, banana<br>• 2x slices grain bread, 1tsp hummus, ½ small tomato, 10g onion, 1/2 cup spinach/rocket	• 2x slice whole meal bread, 30g turkey, 1.5c lettuce, 0.5c salad items (tomato, onion, cucumber), 1tsp reduced fat monounsaturated spread<br>• 2small lamb chops (~92g) cooked in olive oil, 2c salad greens (lettuce, spinach, tomato, cucumber), 0.25c brown rice, 15g fat free French dressing	• 80g salmon fried in olive oil, 0.75c brown rice cooked, 0.75c carrot, ½ potato and 0.6c carrot boiled, 1tb tartare sauce<br>• 1c whole meal pasta, 65g mince beef cooked in olive oil, 0.5c kidney beans, 0.75c passata, ½ carrot, 1c spinach leaves and 20g reduced fat mozzarella<br>• 0.25c couscous cooked in water, 0.75c broccoli, ¼ medium carrot fried in oil with 1 small chicken breast (~113g), 20g reduced fat fetta crumbled on top	• Coffee with air-popped popcorn (salt reduced), 15g pumpkin seeds<br>• 1c yoghurt with 10g pumpkin seeds<br>• 2tbsp hummus, 1 small carrot and ½ small cucumber
15F	• 45g Weetabix (~3 biscuits), 1.5c reduced fat milk, 25g pumpkin seeds, 1 banana, pinch cinnamon<br>• 0.6c rolled oats, 1c reduced fat milk, 1 banana, 25g pumpkin seeds, pinch cinnamon	• 2x slice grain bread with 50g shredded/roast chicken breast, 10g reduced fat mozzarella cheese, 1.5c lettuce, onion, 1tb hummus<br>• Whole meal pita bread, 20g reduced fat mozzarella cheese, 1.5c lettuce/spinach, onion, 1tb eggplant dip	• 50g salmon fried in olive oil, 0.5c brown rice, 0.75c carrot and 1c broccoli steamed, 1tb chili sauce<br>• 1c whole meal pasta, 55g beef mince fried with 0.25c kidney beans, 0.75c passata, ½ small carrot, 100g frozen mixed	• Fruit and nut bar with 220ml orange juice<br>• 25g air-popped popcorn (reduced salt), 1tb eggplant dip, 100g cucumber<br>• 28g almonds, 1c yoghurt, hot chocolate (1tb drinking chocolate powder reduced

		• 1 grain or whole meal wrap (~71g), 70g tuna, 1tsp tartare sauce, 1c lettuce, 0.5c spinach/rocket, onion, cucumber	vegetables 20g mozzarella cheese reduced fat<br>• 0.5c couscous cooked in water, 155g broccoli fried with 95g chicken breast and ¼ medium carrot, 20g reduced fat fetta cheese crumbled on top	sugar + 0.25c reduced fat milk)<br>• 25g air-popped popcorn, 1 orange<br>• 1c yoghurt, 4 brazil nuts
7M	• 0.5c rolled oats, 1c reduced fat milk, 1 banana, 15g pumpkin seeds, 10g almonds, drizzle honey<br>• 2x slices grain bread toasted with 1tsp reduced fat monounsaturated spread, 1tsp vegemite and 1 banana<br>• 1x slice grain bread toasted with 25g avocado, 2 eggs, 1.5c spinach and 1 small tomato	• 2x slices grain bread, 15g turkey, 10g reduced fat mozzarella cheese, 1c lettuce, onion, 2tb hummus, 60g tomato<br>• Grain bread roll (~52g), 70g tuna, 10g reduced fat cheese, 1c lettuce, onion<br>• Whole meal pita bread, 1tb hummus, 25g turkey, 15g reduced fat cheese, 1c lettuce, onion	• 50g fried salmon, 0.25c brown rice, 0.75c steamed carrot and broccoli, 1tb tartare sauce<br>• 0.5c whole meal pasta, 55g beef mince fried in oil with 0.5c kidney beans, 0.5c passata, 10g reduced fat mozzarella cheese, ½ small carrot, 50g spinach<br>• 0.25c couscous, 1c broccoli, 85g chicken breast, 10g reduced fat fetta cheese crumbled on top<br>• 1 small lamb chop (~46g) fried in olive oil, 1c lettuce, 1 tomato, olives, 0.25c cucumber, 5g French dressing	• Medium apple (~164g) and fruit and nut bar<br>• Hot chocolate (1tsp drinking chocolate powder reduced sugar + 1c reduced fat milk)<br>• 200ml orange juice, 1 slice grain bread, 10g peanut butter (reduced sugar and salt)<br>• 1 multigrain rice cake or crispbread, 5-10sg peanut butter

Table S7. Example meals for MedDiet HFB

	Breakfasts	Lunches	Dinners	Snacks
70F	• Omelet: 2x eggs with 70g tomato and mushroom, 10g fetta cheese, 100ml milk fried in EVOO with 2x slices of bread<br>• Peanut butter on 2x slices of multigrain bread<br>• 180g Greek yoghurt with honey, 1 banana<br>• 3 weetabix with 1.25c milk, honey and 1 banana	• 150g sardines on cruskits with 15g mayonnaise<br>• White bean soup served with 1 slice of bread drizzled with 1tb EVOO<br>• Grain flat bread with 0.25c passata sofrito sauce, 20g lean leg ham, 1.5c vegetables (0.5c mushrooms, 30g onion, 0.37c capsicum), 30g mozzarella cheese	• Zucchini patties serves with 1.5c salad vegetables and 1tb EVOO<br>• 65g white fish served with roast veg (40g pumpkin and 120g broccoli)<br>• 1c whole meal pasta with 50g chicken breast fried in 2tb EVOO, 0.25c passata sofrito sauce with 0.75c steamed broccoli	• 150g Greek yoghurt with honey<br>• Tea with 2x biscuits (i.e., yoyo)<br>• 30g mixed nuts<br>• 25g Swiss cheese or ricotta with multigrain crackers<br>• 40g hummus with cucumber and carrot sticks (~100g)
70M	• Omelet: 2x eggs with 70g tomato and mushroom, 10g fetta cheese, 100ml milk fried in EVOO with 2x slices of bread<br>• 4x Weetabix with 1.25c milk, honey and 1x banana	• 150g sardines on cruskits with 15g mayonnaise<br>• Grain flat bread with 0.25c passata sofrito sauce, 0.25c butter beans, 1.5c vegetables (0.5c mushrooms, 30g onion, 0.37c capsicum), 30g mozzarella cheese	• Zucchini patties serves with 1.5c salad vegetables and 1tb EVOO, 0.5c quinoa<br>• 80g trout or salmon with roast vegetables (40g pumpkin, 100g broccoli) roasted in 2tb EVOO<br>• 1c whole meal pasta with 50g chicken breast fried in 2tb EVOO, 0.25c passata sofrito	• 120g Greek yoghurt, 10g pumpkin seeds, honey<br>• 30g Swiss cheese or ricotta with multigrain crackers<br>• 0.75c mashed butter beans with cucumber and carrot sticks (~60g)

			sauce with 0.75c steamed broccoli	
44F	• 4x Weetabix with 1.5c milk, honey, 10g walnuts and banana<br>• 2x slices of multigrain bread, 20g eggplant dip, 1x tomato and spinach<br>• Smoothie with 120g yoghurt, 0.5c fruit, 1.5c milk and 2x Weetabix	• Whole meal flat bread pizza with 1/2c capsicum, 20g cheese, 2tb EVOO, 1/2c passata, 50g shredded turkey<br>• Turkey wrap with hummus and 1.5c mixed salad vegetables<br>• Lentil soup<br>• Lasagna	• 140g tuna on 2x slices bread with onion and spinach<br>• 70g fish fried in EVVO with 2cups mixed vegetables (broccoli, cauliflower, asparagus, spinach)<br>• 1c wholemeal pasta with 60g beef mince fried in 2tb EVOO, 1/4c passata, 10g cheese 1cup vegetables	• 120g yoghurt with 0.75c fruit<br>• 20g eggplant dip with carrot and cucumber sticks<br>• 120g yoghurt and 20g mixed nuts
44M	• 4x Weetabix with 1.5c milk, 20g walnuts, 10g sunflower seeds<br>• Peanut butter on multigrain toast<br>• 120g Greek yoghurt, 1.25c mixed berries, 1.25c milk, 2x weet-bix<br>• 2x eggs, 1tb EVOO to fry tomato and mushroom, 2x slices rye bread	• Whole meal flat bread pizza with 1/2c capsicum, 20g cheese, 2tb EVOO, 1/2c passata, 50g shredded turkey<br>• Lentil soup<br>• Lasagna	• 140g sardines on 2x slices bread with onion and 1.5c spinach<br>• 70g fish fried in EVVO with 2cups mixed vegetables (broccoli, cauliflower, asparagus, spinach)<br>• 1c wholemeal pasta with 65g beef mince fried in 2tb EVOO, 1/4c passata, 10g cheese 1cup vegetables	• 80g hummus with cucumber and carrot sticks<br>• 30g nuts/dried fruit<br>• 120g Greek yoghurt with 1c fruit<br>• 15g pumpkin seeds and orange<br>• 1tin tuna on multigrain crackers

15F	• 4x Weetabix with 1.5c milk, honey and 30g pumpkin seeds<br>• 20g Peanut butter, banana and cinnamon on 2x slices of toast<br>• 170g Greek yoghurt with honey, 1c fruit and 1.5c milk<br>• 2x eggs in 1tb EVOO, 20g cheese, 100g fried vegetables (mushroom, tomato, zucchini)	• 2x slices of rye bread with 1tb basil pesto, 50g chicken, 2cups salad vegetables and 20g mozzarella cheese<br>• Whole meal flat bread pizza with 1/2c capsicum and mushroom, 50g cheese, 2tb EVOO, 1/2c passata<br>• 50g turkey, 1 mixed grain wrap, 2tb hummus, 15g mozzarella cheese and 2c salad vegetables	• 80g fish in 2tb EVOO with roast veg (60g pumpkin and potato, 100g broccoli)<br>• 140g tuna on 2x slices of bread with 1.5c salad vegetables, EVOO and 15g mayonnaise<br>• 0.75c whole meal pasta, 2tb EVOO, 1/4c basil pesto sauce, 10g mozzarella cheese, 130g broccoli, 1.5 home-made chicken patties	• Hot chocolate (1.5c milk and 1tsp drinking chocolate powder)<br>• 170g greek yoghurt with 1.5c fruit and 1x crushed Weetabix<br>• Air-popped popcorn 30g
7M	• 0.75c rolled oats with 1c milk and 1.5c mixed fruit<br>• Peanut butter and banana on 2x slices of whole meal toast<br>• 3x Weetabix with 1.25c milk, 1c mixed fruit and 10g pumpkin seeds<br>• 1 egg, tomato and mushrooms fried in EVOO with 2x slice of rye bread	• 2x slice whole meal bread, 20g cheese, 50g mince beef 2tb passata<br>• Whole meal flat bread pizza with 1/2c capsicum and mushroom, 50g cheese, 2tb EVOO, 1/2c passata<br>• Cheese and salad mixed grain wrap	• Home-made chicken patties with roasted vegetables (60g pumpkin, 60g potato, 60g broccoli)<br>• 70g tuna on 2x slices of rye bread with onion and spinach, 15g mayonnaise<br>• 50g salmon baked in EVOO, 0.25c each broccoli, cauliflower and asparagus, 15g tomato sauce	• Air-popped popcorn + mandarin<br>• Hot chocolate (1.5c milk and 1tsp drinking chocolate powder)<br>• 100 greek yoghurt with 0.25c mixed berries

Table S8. Example meals for Western Diet food basket

	Breakfasts	Lunches	Dinners	Snacks
70F	• 2x slices white bread with butter and vegemite and 200ml orange juice<br>• 2x slices white bread with butter and strawberry jam and 150ml orange juice<br>• 34g (1 sachet) quick oats, 0.75c regular fat milk and 150ml orange juice	• 2x slices white bread with 150g corned beef, cheese, 0.5c lettuce, 25g onion and margarine<br>• Chicken and vegetable pie (138g) with 0.25c frozen peas carrot and corn<br>• 2.5 (97g) fried eggs in butter on 2x slices white bread with margarine<br>• 200g beef lasagna (commercial) with 0.25c each snow peas and carrot steamed	• 2.5x beef sausages regular fat, 0.5c boiled potato and 0.75c green beans with 0.25c gravy and 250ml lemonade<br>• 150g crumbed fish cake with 70g oven roasted potato chips, 0.5c steamed broccoli and 25g garlic aioli<br>• 0.75c pasta with 1c creamy pasta sauce,15g parmesan cheese<br>• 85g fried beef mince on a bread roll with margarine, cheese, lettuce, tomato and cucumber	• Tea/coffee with 2x Anzac biscuits<br>• 90g sticky date pudding with 99g ice cream (1.5 scoops)<br>• Tea/coffee with chocolate chip muffin<br>• 4x wafer style biscuits with 20g cucumber and 42g cheese and 200ml orange juice
70M	• 2x slices white bread with butter and vegemite and 220ml orange juice<br>• 2x slices white bread with butter and strawberry jam<br>• 2x slices white bread with butter and honey	• 2x slices white bread with 210g corned beef, cheese<br>• Chicken and vegetable pie (183g)<br>• 3 (117g) fried eggs on 2x slices white bread with margarine	• 2.5x beef sausages regular fat, 0.25c boiled potato and 0.25c green beans with 0.25c gravy and 250ml lemonade<br>• 200g crumbed fish fake with 90g oven roasted potato chips, 0.25c	• Tea/coffee with 1 small Anzac biscuit and 20g mixed nuts<br>• Tea/coffee and 40g packet potato chips

	• 2x slices white bread with butter and honey with 0.75c canned fruit salad	• 400g beef lasagna (commercial) with 90g oven roasted potato chips	steamed broccoli and tsp garlic aioli<br>• 1c pasta with 1.25c creamy pasta sauce,20g parmesan cheese<br>• 100g fried beef mince on a bread roll with margarine, cheese	• 90g sticky date pudding with 66g ice cream (1 scoop)
44F	• 2x slices white bread with butter and vegemite<br>• 2x slices peanut butter and margarine on toast with an orange<br>• Corn flake breakfast cereal ½c with 1c milk<br>• 2x slices white bread with margarine and jam and an orange	• 2x slices of white bread with margarine, regular fat cheese and 2x slices roast beef<br>• Ham and cheese pizza<br>• Sausage roll<br>• 2x slices white bread with 70g pepperoni, full fat cheese and margarine	• 120g chicken kiev with 80g oven baked potato chips<br>• Chicken breast (85g) roll with butter and cheese<br>• Meat lovers pizza<br>• 100g beef rissole with 80g oven roasted potato chips<br>• 65g beef stir fried in canola oil, 80g potato chips oven roasted, 0.25c frozen peas carrot corn	• Orange and a berry muffin<br>• 2 scoops ice cream<br>• Tea/coffee with potato chips<br>• Sweet biscuit with 250ml orange juice
44M	• 2x slices white bread with margarine and tomato<br>• Corn flake breakfast cereal ½c with 1.5c milk<br>• 2x slices bread with butter and vegemite<br>• 2x slices white bread with peanut butter and margarine and 250ml orange juice	• 2x slices of white bread with margarine, regular fat cheese and 2x slices roast beef<br>• Sausage roll<br>• 2x slices white bread with 70g pepperoni, full fat cheese and margarine, onion and tomato	• 120g chicken kiev with 90g oven baked potato chips<br>• Chicken breast (113g) roll with butter and cheese<br>• Ham and cheese pizza<br>• 100g beef rissole with 100g oven roasted potato chips and tomato sauce	• Muesli bar and potato chips<br>• Tea/coffee with sugar and a sweet muffin<br>• 2 scoops ice cream with 120g canned fruit salad<br>• Handful mixed nuts

15F	• 2x slices white bread with margarine and honey<br>• 3x weet-bix with 1c milk and 1 small banana<br>• 3x pikelets with margarine, maple syrup and a banana	• 2x slices of white bread with margarine, regular fat cheese and 1x slice roast beef<br>• Sausage roll and 200ml coca cola<br>• 2x slices white bread with 2x slices pepperoni, cheese and sliced tomato<br>• 4 chicken nuggets (80g) with 1tb tomato sauce and 2 slices white bread	• 131g chicken Kiev with 0.5c mash potato (added butter, cheese, salt, pepper)<br>• Chicken breast (85g) roll with butter and cheese, tomato sauce and sliced tomato<br>• Chicken and bacon pizza with tomato sauce<br>• 85g beef rissole with 80g oven roasted potato chips, ½ a tomato, 200ml coca cola	• 250ml orange juice and a berry muffin<br>• 45g milk chocolate with 2 scoops ice cream<br>• Chocolate snack bar with 200ml coca cola<br>• 80g potato chips and 200ml coca cola or orange juice<br>• 2x scoop ice cream with jube lollies
7M	• 45g rice-based cocoa cereal with 1.25c full fat milk and 1 banana<br>• 2x slices white bread with butter and vegemite<br>• 2x pikelets with 1tsp margarine, 1tsp maple syrup and a banana<br>• 2x slices white bread with jam and margarine	• 2x slices white bread with 2 slices roast beef, margarine, cheddar cheese and tomato<br>• Sausage roll<br>• 2x slices white bread with 2x slices pepperoni, cheese and sliced tomato<br>• 4 chicken nuggets (~80g) with 60g potato chips, 1tb tomato sauce and 200ml coca cola	• 100g chicken kiew with 0.25c mash potato (added butter, cheese, salt, pepper)<br>• Meat lovers pizza (~100g or ¼)<br>• Chicken breast (30g) roll with margarine, cheese and sliced tomato<br>• 60g beef rissole with 60g oven roasted potato chips	• Chocolate puffed rice bar with 250ml orange juice<br>• 1 scoop ice cream with 2tb chocolate sauce<br>• Berry muffin with 250ml orange juice
